# MyomiRs Expression in Limb Girdle Muscular Dystrophy

**DOI:** 10.1002/iub.70068

**Published:** 2025-10-09

**Authors:** G. Breveglieri, M. T. Altieri, M. T. Rodia, R. Costa, F. Frabetti, G. Cenacchi, G. Sabbioni, M. Borgatti

**Affiliations:** ^1^ Department of Life Sciences and Biotechnology Ferrara University Ferrara Italy; ^2^ Department of Biomedical and Neuromotor Sciences, Cellular Signaling Laboratory, Anatomy Centre Alma Mater Studiorum University of Bologna Bologna Italy; ^3^ Department of Medical and Surgical Sciences Alma Mater Studiorum University of Bologna Bologna Italy

## Abstract

This manuscript is a comprehensive review focused on the role of microRNAs (miRs)—short RNA molecules—in Limb Girdle Muscular Dystrophy (LGMD). LGMD encompasses various and heterogeneous rare genetic neuromuscular diseases, characterized by the progressive wasting and deterioration of muscle fibers, predominantly affecting the pelvic and shoulder girdles. Similar to other muscular dystrophies, LGMD exhibits a dysregulated expression of miRs that are crucial for gene expression regulation and cellular processes. Notably, myomiRNAs, which are preferentially expressed in muscle tissue and linked to muscle cell proliferation and differentiation, appear to be particularly affected. Numerous studies have aimed to identify differentially expressed miRNAs in both physiological and pathological conditions with different purposes: (a) the identification of molecular markers for diagnostic and prognostic purposes, and for evaluation of the effects of possible therapeutic strategies; (b) the detection of a molecular signature to differentiate both LGMD from other muscular dystrophies and LGMD subtypes from each other. The main conclusions so far emerged from published studies are: (a) a high number of differentially expressed miRs have been found in both the serum and muscle fibers of LGMD patients (canonical myomiRNAs, including miR‐1, miR‐133a/b, and miR‐206, are frequently found to be dysregulated across various LGMD subtypes); (b) circulating levels of miR‐206 were found to be significantly elevated in LGMD patients compared to healthy subjects and have been suggested as a potential biomarker of general muscle damage in various muscular dystrophies; (c) possible identification of subtype‐specific molecular signatures (for instance, the combination of six specific miRs has been proposed to discriminate LGMD patients from controls and to identify particular LGMD subtypes, such as LGMDR1, LGMDR2, LGMDR3, and LGMDR4); (d) currently not validated miRNA biomarkers have been described for clinical use yet in LGMD due to heterogeneity of published studies (regarding the type of biological material and techniques used) and limited number of involved patients. Therefore, while miRs show great promise for improving the molecular understanding, stratification, and management of LGMD patients, further rigorous research and validation in larger, standardized patient cohorts are necessary to confirm the clinical reliability of these identified miRNAs.

## 1 Introduction

Non‐coding RNA molecules have been found to play crucial roles in key cellular activities such as cell growth, development, and differentiation, acting in both the nucleus and cytoplasm [1]. Their effects have been studied in physiological conditions and in muscle pathologies that recapitulate the development and differentiation processes of muscle cells [2]. Specifically, microRNA (miRNA or miR) levels were found to be dysregulated in muscular dystrophies, both within muscle fibers and circulating in serum [3], as well as in limb girdle muscular dystrophies (LGMDs) [4, 5]. Therefore, there is increasing interest in studying miRs to clarify the molecular mechanisms underlying these diseases and to evaluate their severity and progression, ultimately aiming to identify new biomarkers [6].

## 2 MiRNAs

**FIGURE 1:**
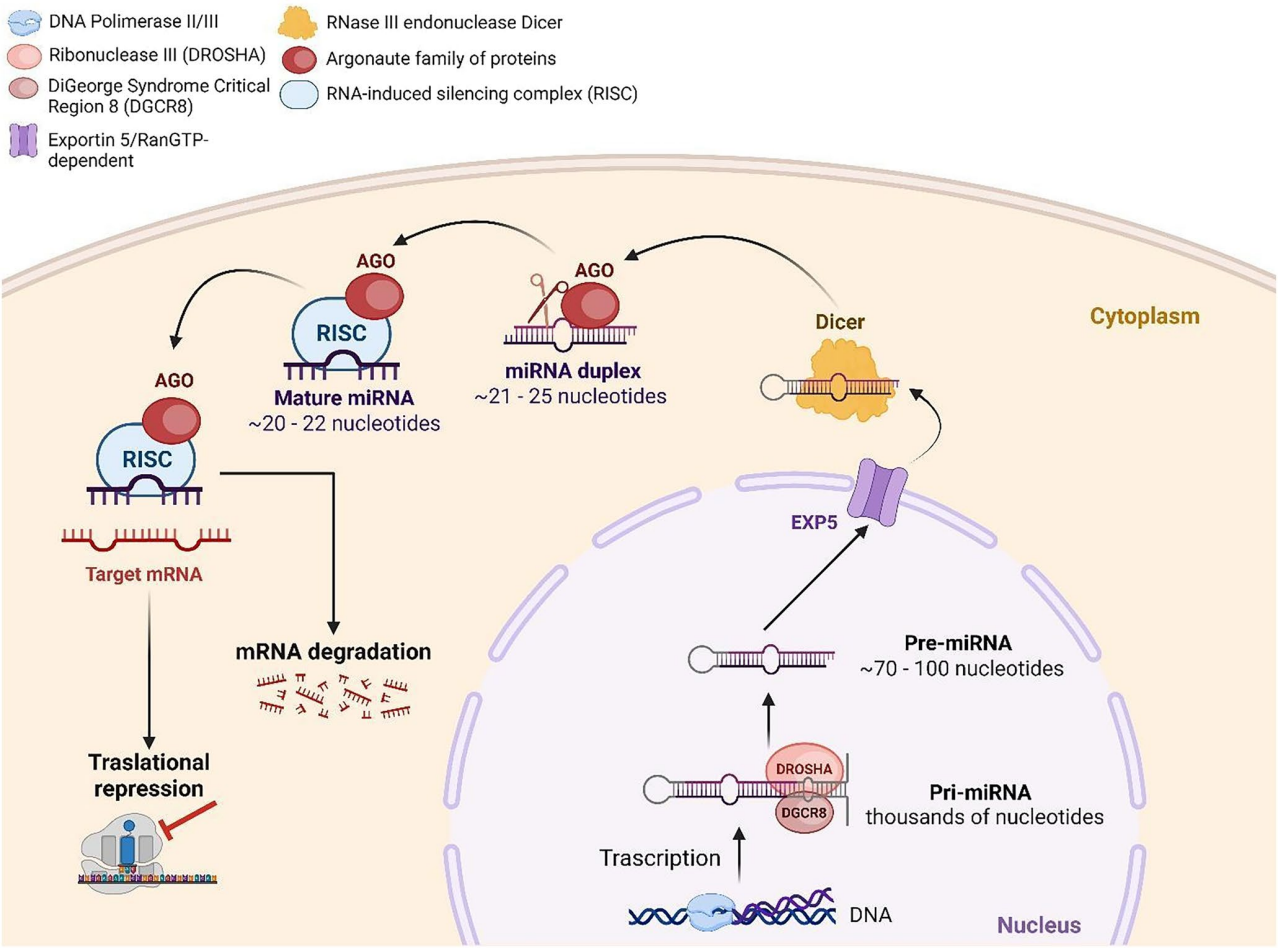
Biogenesis of miRNAs. The process begins with the transcription of primary miRNAs (pri‐miRNAs) by RNA polymerase II in the nucleus. These pri‐miRNAs are processed into precursor miRNAs (pre‐miRNAs) by the Drosha complex. The pre‐miRNAs are then exported to the cytoplasm via Exportin‐5. In the cytoplasm, Dicer, an RNase III enzyme, cleaves the pre‐miRNA to generate a mature miRNA duplex. One strand of the duplex, the guide strand, is loaded onto the RNA‐induced silencing complex (RISC), while the passenger strand is typically degraded. The mature RISC‐miRNA complex regulates gene expression post‐transcriptionally by binding to complementary target mRNAs, leading to translational repression or mRNA degradation. Created in BioRender. Borgatti, M. (2025) https://BioRender.com/5dqop03.

MiRNAs are small, non‐coding RNA molecules, first discovered in *C. elegans*. These molecules regulate gene expression by directly binding to complementary messenger RNA (mRNA) targets within their 3′ untranslated regions (UTRs) as anti‐sense molecules [7, 8] (Figure 1). MiRNA biogenesis begins with transcription by RNA polymerase II or III, producing primary miRNAs (pri‐miRNAs) that become pre‐miRNAs (precursor miRNAs), with a looped hairpin structure, after being cleaved in the nucleus by the Drosha/DGCR8 complex. Pre‐miRNAs are subsequently exported to the cytoplasm by RAN‐GTP/Exportin 5 and further processed by the RNase III endonuclease Dicer complex into short, 20–22 bp duplexes. These miRNA duplexes are then integrated into the RNA‐induced silencing complex (RISC). Within the RISC, one strand (the guide strand) is utilized by the Argonaute (Ago) subunit to identify complementary target mRNAs, while the other strand (the passenger strand) is degraded [8, 9]. Depending on the degree of complementarity between the miRNA and its target mRNA, the mRNA will either be degraded or translationally repressed, thereby preventing its expression and function [8].

Mammalian miRNAs are identified and classified according to their sequence homology with already known miRs from *C. elegans* [10]. As both miRNA strands can sometimes target different mRNAs, miRNAs originating from the 5′‐or 3′‐end of the pre‐miRNA hairpins are now designated as −5p and −3p miRNAs, respectively [11]. Furthermore, the prediction of mammalian miRNA target genes is possible using specialized software and computational methods [12].

MiRNAs are released by various cell types and exhibit stability in the bloodstream thanks to their ability to circulate via exosomes [10]. Their expression levels are often altered in pathological conditions compared to physiological ones [13]. Indeed, miRNAs play crucial roles in regulating protein functions in several conditions such as cardiac function, immune regulation, and cancer [14]. Consequently, their levels are frequently dysregulated in conditions like cancer and neuromuscular, metabolic, and cardiovascular diseases [15, 16, 17, 18]. Given their stable circulation in the bloodstream, miRNAs have been proposed as promising biomarkers [19, 20] for the diagnosis, monitoring, or prognosis of different disorders and conditions, including neuromuscular diseases [21].

## 3 Myogenesis

Myogenesis is a multi‐step process consisting of the generation and development of muscular tissue from undifferentiated cells, where myoblasts proliferate and differentiate before and after birth [22]. Briefly, somitic stem cells generate myogenic stem cells expressing Pax3 and Pax7, which are myogenic precursors. Some of them will differentiate into proliferating myoblasts, merging into multinucleated myotubes in the terminal differentiation phase. Finally, myotubes generate mature myofibers, parallel aligned. Instead of satellite cells, allowing adult (postnatal) skeletal muscle to regenerate, they originate from myogenic precursors: after activation, they generate myoblasts as well [9].

Myogenic stem cells proliferate and differentiate according to a critical regulation by transcription factors acting in cascade, whose expression mode is in agreement with their role and regulation. In particular, the gene family of myogenic regulatory factors (MRFs) includes Myf5, MyoD (myoblast determination gene number 1), Myogenin (MyoG or Myf4), and Mrf4 (Myf6, also called Herculin), whose sequences and roles are conserved [9].

All MRFs have myogenic ability that can turn fibroblasts into myoblasts [9]. Their activation occurs sequentially during embryogenesis, with a specific expression pattern. For example, in mouse embryogenesis, the first expressed MRF is Myf5 [23], followed by Myogenin [24], whereas the expression of Mrf4 and MyoD occurs only later [25, 26]. The Mrf4 transcript levels are increased after birth when it becomes the leading MRF [25]. The specificity of MRF gene expression indicates that it may have different functions during the different myogenesis phases, with the aim of inducing myogenic gene expression [9].

The interactions and regulations of myogenic regulatory factors have been extensively reviewed by Chen et al. [9], who listed all miRNAs implicated in myogenesis as well.

In recent years, the importance of all MRFs for myogenesis has been demonstrated, together with the miRNA regulation of their mRNAs [9].

## 4 Muscle‐Specific microRNAs (myomiRNAs)

The characterization of molecular pathways controlled by miRNAs has revealed their involvement in all stages of myogenesis, where they act synergistically or antagonistically and in close association with myogenic factors [27].

MiRNAs preferentially found in skeletal muscles and/or in cardiac muscles, are termed myomiRNAs [28] or myogenic miRNAs as they are potent activators of myogenesis [9]. Canonical myomiRNAs, considered muscle‐specific, belong to the miR‐1, miR‐206, and miR‐133 families, are found in both cardiac and skeletal muscle [29] and are involved in regulating main muscle processes such as myogenesis, muscle remodeling, and muscle development [28, 30, 31, 32].

Nowadays the term “myomiRNA” also indicates several muscle‐enriched miRNAs encoded within myosin genes, such as miR‐499 and miR‐208, in addition to miRNAs involved in the development of cardiac muscle [33]. So, currently eight miRNAs can be considered myomiRNAs: miR‐1, miR‐133a, miR‐133b, miR‐206, miR‐208a, miR‐208b, miR‐486, and miR‐499 [22, 34, 35, 36]. All myomiRNAs can be found both in cardiac and in skeletal muscle, except for miR‐206, which is specific for skeletal muscle, and miR‐208a, which is specific for cardiac muscle [28]. They can also be detected in other tissues, even though they preferentially act on muscle [37], like miR‐486 that can also be defined “muscle‐enriched” [34, 38].

In humans, canonical myomiRNAs are encoded by genes located in bicistronic clusters on human chromosomes [39]:

miR‐1‐1/miR‐133a‐2: chromosomal region 20q13.33; intragenic, located in an intron of the *C20orf166* (chromosome 20 open reading frame 166) gene;

miR‐1‐2/miR‐133a‐1: chromosomal region 18q11.2; intragenic, located in an intron of the *MIB1* (mindbomb E3 ubiquitin protein ligase 1) gene;

miR‐206/miR‐133b: chromosomal region 6p12.2; intergenic.

Consequently, some myomiRNAs are transcribed together. Even when genomic regions differ, mature miRNAs exhibit high sequence similarity.

Moreover, canonical myomiRs are evolutionarily and genomically linked [27, 28]. For example, there are no sequence differences between miR‐1‐1 and miR‐1‐2 that have the same seed sequence of miR‐206 and are different from it by 4 nucleotides in the 3′ region; also, miR‐133a‐1 and miR‐133a‐2 have the same sequence and are different from miR‐133b by 2 nucleotides. On the contrary, some myomiRNAs are monocistronic and their sequences are sited in the coding sequence of specific genes: for example, miR‐208a, miR‐208b, and miR‐499 are found in the intronic sequences of the myosin heavy chain genes MYH6, MYH7, and MYH7B, respectively [40], whereas miR‐486 is located in the intron of the ANK1 (ankyrin 1) gene [34].

The role of myomiRNAs and other miRs in muscle during myogenesis has been reviewed [41], together with their functions and implications in exercise, atrophy, aging, and diseases [15, 22, 42, 43, 44]. It is known that they are involved both in embryonic and in adult myogenesis [13]. Briefly, during myoblast differentiation there is an increase of miR‐1 and miR‐206 [30, 45], which have anti‐proliferative effects. Their expression begins the second differentiation day of myoblasts, promoting differentiation and blocking proliferation. These two miRNAs are crucial for correct myoblast differentiation and muscle development [46].

The regulation of myomiRNAs expression, having an active role during myogenesis, has been extensively reviewed by Horak et al. in 2016 [28], as well. In particular, MRFs seem to be involved in crucial feedback mechanisms [30, 36, 47], together with other factors like MYOD1, MYF5, MYF6, and MEF2, MYOG (myocyte enhancer factor 2), MKL1 (megakaryoblastic leukemia (translocation) 1), SRF (serum response factor), and other transcription factors associated with muscle [27, 34, 48, 49, 50]. In detail, as reviewed by Costa et al. in 2022 [5], MyoD and IGF‐1 (insulin‐like growth factor 1) activate miR‐1 and miR‐206, both downregulating Pax3 and Pax7. So, genes determining MyoD and Myf5 upregulation are activated, and cell cycle block and proliferation arrest occur, promoting commitment and proliferation of myoblasts. In addition, the transcription repressor of many muscle genes like MyoG and MEF2, histone deacetylase 4 (HDAC4), is inhibited by miR‐1 and miR‐206, promoting myoblast differentiation towards myotubes. Also, miR‐133a and miR‐133b inhibit myoblast proliferation and stimulate differentiation by controlling MAPK signaling and miR‐133 expression, whose upregulation is produced by IGF‐1 by inducing MyoG; as a result, a negative feedback process occurs, resulting in myofibers maturation [28].

Also other not muscle‐specific miRNAs have found actively involved in muscle development, such as miR‐26a [51], miR‐27 [52], miR‐181 [53], miR‐221 and miR‐222 [54, 55], miR‐322/miR‐424 and miR‐503 [56], in addition to miR‐24, miR‐29, miR‐125, miR‐214, miR‐675 and many others [22, 38], whose number is progressively increasing.

Furthermore, miRNAs regulate myogenic stem cells (or satellite cells) activation and quiescence. In the adult, these cells are found between basal lamina and the myofiber. Their activation, generating proliferating progenitor cells aimed at regenerating damaged skeletal muscle, is a consequence of damage signals [9]. For example, among the 412 detected miRNAs by Castel et al. in 2018 [57] in myogenic stem cells, 249 miRNAs were differentially expressed in activated, differentiated, and quiescent cells.

As a consequence of MRF involvement, a lot of skeletal muscle disorders are characterized by a deregulation of miRNA expression [3, 58, 59], generally including myomiRNAs. That's what happens in muscular dystrophies, as studied by Ballarino et al. in 2016 [27], where alterations of circulating miRs have also been documented [4, 60].

Obviously, studying miRNA levels in muscular dystrophies can help to understand their effect on myogenesis both in pathological conditions and in physiological ones. Additionally, advances in identifying new molecules involved in the regeneration and differentiation of muscle could allow us to understand the molecular processes causing these disorders and to identify new potential therapeutic targets.

## 5 Muscular Dystrophies

Muscular dystrophies are a class of rare and heterogeneous neuromuscular disorders. They share common clinical and pathological features, including progressive weakness, muscle wasting and deterioration, with gradual loss of muscle tissue, necrosis, muscle fiber regeneration, increased fibrosis and adipose tissue substitution [61, 62]. The different forms of muscular dystrophies up to now discovered are more than 50, where the clinical phenotype is different according to the principally involved muscles, the weakness level, the onset time and the progression rate [63, 64]. Various genes are implicated in the distinct forms of muscular dystrophies and in the disorder onset, and different pathogenic mutations have been identified as well [65, 66].

The main muscular dystrophies types are reported in Table 1 [60, 67, 68, 69, 70, 71, 72, 73].

**TABLE 1:** Main types of muscular dystrophies. The disease incidence, main features, and affected genes are reported.

Name	Abbreviation	Incidence	Features	Affected gene	References
Duchenne muscular dystrophy	DMD	1 in 3500	The most common and severe muscular dystrophy. Progressive muscle necrosis and wasting, leading to death in early adulthood due to cardiorespiratory failure.	Mutations in the X‐linked dystrophin (DYS) gene, preventing the production of functional dystrophin protein.	Duan et al. [67]
Becker muscular dystrophy	BMD	3–6 in 100,000	The same muscle wasting distribution and weakness of DMD, but more benign disease course.	Mutations in the DYS gene, producing a partially functional dystrophin protein.	Straub and Guglieri [68]
Facioscapulo‐humeral muscular dystrophy	FSHD	4–10 in 100,000 (about 95% FSHD1, 5% FSHD2)	Progressive weakness and atrophy of the skeletal muscles of face, shoulders, arms, legs and abdominal muscles.	Inappropriate reactivation of DUX4 gene. FSHD1: deletion of repetitive elements on DUX4 gene. FSHD2: mutations in SMCHD1 gene.	Salsi et al. [69]
Myotonic dystrophy	DM	DM1: 1 in 8000 DM2: 0.99 in 100,000	DM1: variable onset from congenital to very late‐onset (most common); distal predominant muscle weakness; cardiac, respiratory, and central nervous system involvement. DM2: adult‐onset or late‐onset; proximal predominant muscle weakness; less severe multisystem involvement.	DM1: CTG repeat expansion in the DMPK gene. DM2: CCTG repeat expansion in the CNBP gene.	LoRusso et al. [70]
Limb girdle muscular dystrophy	LGMD	1:100,000	Progressive weakness and wasting of pelvic, scapular and trunk muscles.	More than 30 genes involved. For example, calpain3 (CAPN3) gene (calpainopathy), dysferlin (DYSF) (dysferlinopathy), sarcoglycan (SGCA, SGCB, SGCG and SGCD) genes (sarcoglycanopathies), transportin‐3 (TNPO3) gene (transportinopathy).	Johnson and Statland [71]

The patient's identification generally relies on histopathological analysis, clinical phenotype, and genetic analysis [64], but also misdiagnoses frequently occur because of the similarity of clinical symptoms. As in most diseases, miRNAs take part in the cellular reaction to deregulated proteins; an improvement of diagnosis, prognosis, or treatment follow‐up for muscle disorders could be achieved in these patients by identifying specific disease‐associated miRNAs.

## 6 Muscular Dystrophies and myomiRNAs

A demonstration of miRNA expression deregulation in different human neuromuscular disorders was provided by a large‐scale miRNA analysis described by Eisenberg et al. in 2007 [3]. The study was aimed at finding muscle‐specific miRNAs potentially associated with Duchenne Muscular Dystrophy (DMD), Facioscapulo‐Humeral Muscular Dystrophy (FSHD), or Limb Girdle Muscular Dystrophy (LGMD) diseases. In particular, considering 10 distinct primary muscular diseases and analyzing muscle biopsies by microarray technique, a total of 185 miRNAs were described that are up‐ or down‐regulated, demonstrating a differential miRNA profile. Among them, 151 miRNAs resulted up‐regulated and 28 miRNAs down‐regulated compared to control, while six miRNAs (miR‐30b, miR‐92, miR‐361, miR‐423, miR‐29a, and miR‐29b) were found only in some diseases and without a specific expression pattern. In all the analyzed samples, two miRNAs (miR‐146b and miR‐221) resulted dysregulated, while miR‐155, miR‐214, and miR‐222 were found differentially expressed in all diseases except Becker Muscular Dystrophy (BMD). Instead, some other miRNAs were differentially expressed only in one of the analyzed diseases: miR‐22, miR‐26a, miR‐30a‐5p, miR‐30d, miR‐30e‐5p, miR‐95, miR‐101, miR‐193b, miR‐331, miR‐485‐5p, miR‐486, ambi‐miR‐7075, and ambi‐miR‐13156 in DMD; miR‐517* in FSHD; ambi‐miR‐10,617 in LGMD2A; miR‐301 in LGMD2B; miR‐302c* in Miyoshi Myopathy (MM).

More recently, a review by Zacharewicz et al. [74] focused on a specific group of miRNAs implicated in muscle development, function, and disease, indicating the following miRs, the so‐called “dystromiRs”, as dysregulated in most neuromuscular diseases: miR‐1, miR‐21, miR‐31, miR‐133a, miR‐142‐3p, miR‐149‐5p, miR‐193b‐3p, miR‐206, miR‐378a‐3p [13]. Moreover, the expression of some of these dysregulated miRs was found to be disease‐specific [13]. In addition, as miRNAs can circulate in the bloodstream through exosomes [10], some serum miRNA levels seem to be altered in various neuromuscular diseases, resulting in a promising possible diagnostic signature specific for each mutation and for specific neuromuscular diseases [13, 75, 76, 77].

For example, the small RNA sequencing of plasma from patients and healthy subjects indicated that levels of miR‐19b‐3p, miR‐122‐5p, miR‐192‐5p, miR‐206, and miR‐323b‐3p were able to distinguish LGMD, DMD, and FSHD patients from controls, with a specific expression pattern according to the disease [13].

In DMD, one of the most investigated neuromuscular disorders, an increase in the canonical myomiRNAs (miR‐1, miR‐133a, and miR‐206) levels, both in skeletal muscle biopsies and in serum, was reported after analysis by real‐time quantitative PCR (qPCR) or droplet digital PCR (ddPCR) [75, 76, 78, 79]. The same increase was found in DMD animal models, including mouse models not producing dystrophin and the CXMDj (canine X‐linked muscular dystrophy) Japan dog, respectively [75, 80, 81, 82, 83]. Dystrophin plays a crucial role in muscle differentiation by controlling the switch from the early to the late stages of the process, by means of a pathway comprising neuronal NOS (nNOS) and histone deacetylase 2 (HDAC2) [84]. Some miRNAs implicated in muscle terminal differentiation, such as miR‐1 and miR‐133, and the more ubiquitous miR‐29 and miR‐30, were identified as targets of this pathway [85]. In dystrophic muscles, the dysregulation of these miRs is correlated with dystrophic pathogenesis [40, 85]. On the contrary, miR‐206 is not involved in the dystrophin/nNOS/HDAC2 pathway. Its levels are low in adult myofibers, whereas they increase during DMD muscle regeneration [85]. Its deletion made the dystrophic phenotype in mdx mice more severe [86], while miR‐206 over‐expression stimulated satellite cell differentiation and fusion to generate new myofibers when the disorder causes damage to the existing ones [27].

Other miRNAs were found dysregulated in serum or skeletal muscles of DMD patients, as miR‐21, miR‐29, miR‐31, miR‐199a, miR‐208b, miR‐486, miR‐499 [10]. In particular, miR‐31, controlling satellite cell activation and regulating fiber maturation, shows high levels in pathological muscles, where it is involved in a delay of muscle differentiation [87]. Interestingly, the dystrophin rescue resulted increased in DMD myoblasts by miR‐31 inhibition, after exon skipping treatment [27]. Koutsoulidou and Phylactou, in 2020, described other miRNAs that sporadically seem to be involved in DMD [21]. The more recent review by Kiełbowski et al. [76], addressing microRNA alterations in DMD, summarizes the most recent studies about miRNA dysregulation in DMD patients.

Furthermore, in DMD patients, a correlation was found between the levels of specific myomiRNAs and the severity and progression of the disease [75, 76], suggesting a possible use of miRNAs as novel monitoring biomarkers for DMD.

Also in the serum of BMD patients, an increase of myomiRNAs (miR‐1, miR‐133, miR‐206) levels was reported in comparison to controls, even if it resulted lower than in DMD patients [75].

As regards other muscular dystrophies, RT‐qPCR miRNA analysis of primary skeletal muscle myoblasts derived from Myotonic Dystrophy type 1 (DM1) and type 2 (DM2) patients revealed specific diagnostic signatures of dysregulated microRNAs [88, 89], suggesting them as potential biomarkers also for DM. In particular, in the case of the most frequent form in adult people, DM1, miR‐1 and miR‐335 resulted up‐regulated, while miR‐29b, miR‐29c, and miR‐33 were found down‐regulated in DM1 biopsies compared to control subjects, but not in DM2 patients. The only miR seeming to be specifically modulated only in DM1, and not in other muscular disorders, was miR‐33. Instead, miR‐1, miR‐206, and miR‐133b displayed an abnormal cellular localization [88], while their amounts resulted high in the serum of patients and correlated with muscle wasting [90]. In addition, in a wider group of DM1 patients, a reverse transcription (RT)‐qPCR validation of miR‐27b, miR‐140‐3p, miR‐454, and miR‐574 as plasmatic biomarkers for DM1 was described [91].

Instead, in a little cohort of DM2 patients, seven of the miRNAs identified in DM1 patients (miR‐1, miR‐133a, miR‐133b, miR‐140, miR‐206, miR‐454, and miR‐574) resulted dysregulated in plasma [91]. In addition, in DM2 patients, after miRNA array analysis, seven miRNAs were found increased in skeletal muscle tissue in comparison to controls (miR‐34a‐5p, miR‐34b‐3p, miR‐34c‐5p, miR‐146b‐5p, miR‐208a, miR‐221‐3p, and miR‐381), whereas four miRNAs resulted reduced (miR‐125b‐5p, miR‐193a‐3p, miR‐193b‐3p, and miR‐378a‐3p): among them, miR‐193b‐3p, miR‐208a, and miR‐381 were similarly modulated also in DM1 patients [89]. Anyway, more information is needed before suggesting miRNAs as biomarkers for the less common forms of muscular dystrophies like DM2.

Moreover, in FSHD, together with the increased expression of muscle myomiRNAs, other miRNAs were found dysregulated, including miR‐411, overexpressed in FSHD myoblasts [92, 93]. More specifically, Harafuji et al. [92], after identification of 8 dysregulated miRNAs in proliferating FSHD primary myoblasts by microRNA array analysis, validated the up‐regulation of miR‐411 in primary and immortalized FSHD myoblasts in comparison to controls by using qPCR [92]. On the contrary, Dmitriev et al. [93] evaluated miRNA expression in primary myoblasts and myotubes from FSHD patients and healthy subjects by TaqMan Low Density Array (TLDA), identifying a specific miRNA profile of FSHD myoblasts and myotubes. Twenty‐nine total miRNAs were found differentially expressed between patients and controls: 21 miRNAs resulted up‐regulated (miR‐1, miR‐7, miR‐15a, miR‐22, miR‐30e, miR‐32, miR‐107, miR‐133a, miR‐133b, miR‐139, miR‐152, miR‐206, miR‐223, miR‐302b, miR‐331, miR‐362, miR‐365, miR‐382, miR‐532, miR‐654, miR‐660), while 8 were down‐regulated (miR‐15b, miR‐20b, miR‐21, miR‐25, miR‐100, miR‐155, miR‐345, and miR‐594) [93].

A study by Koutsoulidou et al. [63], aimed at identifying novel miRNA in serum as biomarkers for the rarest forms of muscular dystrophies, detected many miRNAs preferentially associated with patients rather than with healthy subjects. In particular, specific serum miRNA signatures were identified in FSHD1, LGMD2A, and DM2 patients thanks to high‐throughput Next‐Generation Sequencing (NGS) of small RNAs. The two best candidate miRNAs for each disease, chosen and validated in a greater group of patients, were miR‐25‐3p and miR‐363‐3p for DM2, miR‐206 and miR‐223‐3p for FSHD1, miR‐143‐3p and miR‐486‐3p for LGMD2A.

A genome‐wide analysis of differentially expressed small RNAs in serum of muscular dystrophy patients and healthy subjects by small RNA NGS was performed by Kakouri et al. in 2022 [72], as well. In patients, an alteration of various miRNAs, already known and previously uncharacterized, was reported, with an average of 76 under‐ and 70 over‐expressed miRNAs. Some of them are common across patients affected by distinct forms of muscular dystrophies, suggesting that they can be found in the patient's blood as a consequence of muscle degradation and not specifically related to the disease. For example, miR‐206 has been revealed as a possible biomarker of muscle damage in various muscular dystrophies [63]. Also, miR‐30c and miR‐181 can be used as biomarkers to monitor muscle wasting, as they have a high expression in muscle, with a similar pattern in various muscular dystrophies. On the opposite, other identified miRNAs are disease‐specific, suggesting a distinctive pattern of circulating miRNAs for the different patient classes, confirming their involvement in the disorders' pathogenesis and also the possibility to find a good biomarker for the different forms of muscular dystrophies [72]. Furthermore, the target genes of the most notable miRNAs were bioinformatically analyzed, identifying distinct pathways involved in the various forms of muscular dystrophy. A total of 418, 486, 490, 826, and 308 genes were predicted as targets for DMD, DM2, DM1, FSHD1, and LGMD R1 calpain3‐related, respectively; in addition, 249 disease‐specific gene targets were found for DMD, 205 for DM2, 252 for DM1, 456 for FSHD1, and 85 for LGMD R1 calpain3‐related [72].

Some miRNAs, including those identified and investigated in muscular dystrophies, are involved in the molecular pathways underlying key clinical and pathological features, such as fibrosis, muscle regeneration, and satellite cell survival. An overview of selected miRNAs that are either regulated or functionally implicated in these three aspects is presented in Table 2 [52, 86, 94, 95, 96, 97, 98, 99, 100, 101, 102, 103, 104, 105, 106, 107, 108, 109, 110, 111, 112, 113, 114, 115, 116, 117, 118, 119, 120, 121]. The functional roles of miRNAs have been explored using various in vivo and in vitro models employing different detection methodologies (e.g., qPCR‐ or NGS‐based approaches) across multiple sample types. Importantly, these clinical features are not independent but interconnected; for instance, muscle regeneration relies on satellite cell activation and survival, whereas fibrosis under physiological conditions can also activate regenerative pathways. Therefore, some miRNAs, such as miR‐24 and miR‐27, are not exclusively associated with a single biological mechanism but rather participate in multiple interconnected processes.

**TABLE 2:** Studies on microRNAs (miRs) and their involvement in key clinical aspects of muscular dystrophies. The clinical aspects, miRs, and detection methodologies are presented.

Clinical aspects	miRs	Methodology	References
Fibrosis	miR‐29b	RNA‐seq dataset re‐analysis (MIENTURNET) and RT‐qPCR validation in muscle tissue sample (murine model)	[94]
		miRNA expression analysis by RT‐qPCR in muscle biopsies sample and in cell cultures (human model)	[95]
	miR‐146a‐5p	miRNA expression analysis by RT‐qPCR in muscle tissue sample and in cell cultures (murine model)	[96]
		miRNA expression analysis by RT‐qPCR in muscle tissue sample (murine model)	[97]
	miR‐1	RNA‐seq dataset re‐analysis (MIENTURNET) and RT‐qPCR validation in muscle tissue sample (murine model)	[94]
	miR‐27b‐3p	miRNA expression analysis by RT‐qPCR in muscle tissue sample (murine model)	[98]
	miR‐199a‐5p	miRNA expression analysis by RT‐qPCR and miRNA array validation in culture of cells derived from muscle biopsies and their exosomes (human model)	[99]
	miR‐24	miRNA expression analysis by RT‐qPCR in muscle tissue sample and in cell cultures (murine model)	[100]
	miR‐21	miRNA expression analysis by RT‐qPCR in muscle tissue sample and in cell cultures (murine and human model)	[101]
		miRNA expression analysis by RT‐qPCR in muscle biopsies sample and in cell cultures (human model)	[95]
	miR‐122	miRNA expression analysis by RT‐qPCR in muscle tissue sample and in cell cultures (murine model)	[100]
Muscle regeneration	miR‐199‐3p	miRNA expression analysis in cell cultures, muscle tissue sample and free circulating miRNAs in blood, plasma and exosome by RT‐qPCR (murine model)	[102]
	miR‐675‐3p/miR‐675‐5p	miRNA expression analysis by RT‐qPCR and miRNA array in muscle tissue sample and in cell cultures (murine and human model)	[103]
	miR‐31	miRNA expression analysis by in situ hybridization and RT‐qPCR in muscle tissue sample (murine model)	[104]
		miRNA expression analysis by in situ hybridization in cells cultures (murine model)	[105]
	miR‐24‐3p	miRNA expression analysis by RT‐qPCR in muscle tissue sample and in cell cultures (murine model)	[106]
	miR‐431	miRNA expression analysis in muscle tissue sample by northern blot and expression of miRNAs in cells cultures by RT‐qPCR (murine model)	[107]
		miRNA expression analysis in cell cultures by NGS and miRNAs in muscle tissue sample by RT‐qPCR (murine model)	[108]
	miR‐200c‐5p	miRNA expression analysis by RT‐qPCR in muscle tissue sample and in cell cultures (murine model)	[109]
	miR‐127	miRNA expression analysis in muscle tissue sample by northern blot and RT‐qPCR. MiRNAs in cells cultures by RT‐qPCR (murine model)	[110]
	miR‐206	miRNA expression analysis by northern blot and RT‐qPCR in muscle tissue sample and cell cultures (murine model)	[86]
	miR‐133b	miRNA expression analysis by RT‐qPCR in muscle tissue sample (murine model)	[111]
	miR‐155	miRNA expression analysis by RT‐qPCR in muscle tissue sample and in cell cultures (murine model)	[112]
	miR‐106b	miRNA expression analysis by in situ hybridization and RT‐qPCR in muscle tissue sample, muscle biopsy sample (murine and human model)	[113]
Satellite cell survival	miR‐378	miRNA expression analysis by RT‐qPCR in muscle tissue sample (murine model)	[114]
	miR‐143‐3p	miRNA expression analysis by RT‐qPCR in muscle tissue sample and cell cultures (murine and human model)	[115]
	miR‐24	miRNA expression analysis by RT‐qPCR in muscle tissue sample and cell cultures (murine and human model)	[116]
	miR‐194‐5p	miRNA expression analysis by RT‐qPCR in muscle tissue sample (rabbit model)	[117]
	miR‐489	miRNA expression analysis using RT–qPCR‐based miRNA arrays in cell cultures (murine model)	[118]
	miR‐195/miR‐497	miRNA expression analysis using miRNA microarray and RT–qPCR analyses in cell cultures (murine model)	[119]
	miR‐27b	miRNA expression analysis by RT‐qPCR and in situ hybridization in cell cultures (murine model)	[52]
	miR‐23b/miR‐27b	miRNA expression analysis by RT‐qPCR and miRNA array in muscle tissue sample and cell cultures (human model)	[120]
	miR‐148a‐3p	miRNA expression analysis by RT‐qPCR in muscle tissue sample and cell cultures (chicken model)	[121]

Finally, dystrophin mRNA also demonstrated to be targeted by the inflammatory miR‐146b, miR‐223, and miR‐374a, increasing in dystrophic muscles according to the severity of the disorder [122]. In particular, the activation of miR‐146b and miR‐223 in dystrophic myofibers depends on inflammation and involves the TNF‐α–induced NF‐κB signaling pathway. All of them are found augmented in different muscle pathologies as well [3, 85], suggesting that inflammation‐associated miRNAs are involved in all muscle diseases characterized by chronic inflammation, and their targeting should be evaluated as a possible therapeutic option for muscular dystrophies in combination with other strategies.

Overall, the interest in the identification of biomarkers for this class of diseases is continuously increasing in order to evaluate both the disorder development and the effect of novel therapies.

## 7 LGMD

LGMDs are a class of rare heterogeneous hereditary neuromuscular disorders sharing a progressive weakness and deterioration of muscle fibers of the shoulder and pelvic girdle [123, 124, 125].

The disease has been found to have an overall incidence of about 1:100,000 [126]. Clinically, there is a great phenotypic variability in terms of involved muscles, weakness degree, onset age, and disease progression. This results in both milder forms, having a late onset and a slow development, as well as more severe forms, having an earlier onset and a quicker progression [4]. Generally, damage to the muscle membrane, to its repair systems, to sarcomere remodeling, to the cytoskeleton, and its interactions with membranes can be found in all the variants [127].

Different alterations have been found in more than 30 genes, each responsible for a distinct LGMD subtype. These mutations contribute to a wide difference not only in genotype but also in phenotype, making it difficult both to diagnose the disease and to make a prognosis or plan a possible treatment [128].

An exhaustive classification of LGMD forms was provided by Bouchard and Tremblay in 2023 [125].

Briefly, LGMDs are classified considering genetic inheritance, the order of discovery, and clinical and histopathological features [4, 129]. The name LGMD is associated with the letter R or D in the case of recessive or dominant disease, respectively, and to a progressive number according to the discovery order. To date, 8 autosomal dominant and 23 recessive subtypes are known [130, 131]. Calpainopathy, dysferlinopathy, sarcoglycanopathies, and transportinopathy are the most common [132].

The calpainopathy (LGMDR1) is a recessive and the most widespread subtype of LGMD, with about 40%–50% of total cases of LGMD in European countries [133]. It is caused by a deficiency of calpain3 (CAPN3), a calcium‐activated cysteine protease specific for skeletal muscle, and essential for muscle regeneration. Calpain‐3 regulates sarcomeric protein turnover and helps to establish self‐renewing reserve cells through the control of MyoD protein level [134, 135, 136, 137]. It comprises two subtypes: G1, where calpain3 is absent, and G2, where calpain3 function is impaired [127].

LGMDR1 displays selective, increasing, and symmetrical weakness of the proximal limb and girdle muscles, especially the ones of the hips and shoulders. The heart is not involved, and there is no intellectual disability. In addition, in patients, calf muscles enlarge, muscles shorten, and harden, causing contractures, and scoliosis and winging of the shoulder blades can be found [138]. According to how muscle weakness distributes at the disease onset, two main subtypes have been described: the most frequently found is the pelvic‐femoral, where muscle weakness starts in the pelvic girdle and then moves to the shoulder girdle; on the contrary, the scapulohumeral subtype generally has a milder phenotype, and muscle weakness occurs first in the shoulder girdle and affects the pelvic girdle only later [139]. Levels of creatinine kinase (CK) are high since childhood, whereas the onset age is different, ranging between 2 and 40 years.

The dysferlinopathy (LGMDR2) shows weakness and atrophy of the pelvic and shoulder girdle muscles, with onset in puberty and slow development [140]. The pathological mutations are in the DYSF gene, coding for dysferlin, a 230‐kDa large transmembrane protein taking part in the membrane repair process, in the intracellular vesicle system and in T‐tubule development in skeletal muscle [141]. Calpain‐3 belongs to the dysferlin complex and has a regulatory function by cutting AHNAK, a protein complex‐associated and implicated in subsarcolemmal cytoarchitecture and membrane repair [127]. LGMDR2 can be considered a calpainopathy as well, because it shows a secondary reduction of calpain‐3 caused by a deficiency of functional dysferlin [127].

Sarcoglycanopathies (LGMDR3‐6) originate from alterations in genes coding for sarcoglycans (SGCA, SGCB, SGCG, and SGCD), muscle‐specific protein subunits of the sarcoglycan complex. This complex is joined to dystrophin in cardiac and skeletal muscle [142], anchors the cellular cytoskeleton to the extracellular matrix [143] and is important in muscle contraction, maintaining muscle membrane stability. Alterations of sarcoglycan genes can make muscle fibers more subject to injuries during muscle contractions [142]. Patients with sarcoglycanopathies show a broad range of clinical phenotypes, including more severe and milder forms, with increasing muscle weakness and atrophy, especially of pelvic and shoulder girdles, together with cardiac and respiratory involvement [138]. On the contrary, no cognitive dysfunctions are present. The onset of muscle weakness starts typically during childhood, and the disorder progresses more rapidly and severely than other LGMDs [144].

The transportinopathy (LGMDD2) is caused by a mutation in the TNPO3 gene, encoding for transportin‐3, a member of the importin beta family whose function is to translocate proteins from cytoplasm to nucleus [145]. Particularly, TNPO3 targets are rich in serine/arginine domains (SR), including proteins implicated in mRNA splicing and metabolism and essential splicing factors [146]. LGMDD2 is rare and dominant, and the pathogenic mutation is the deletion of an adenine in the TAG stop codon of the TNPO3 gene, resulting in a 15 codon lengthening of the reading frame, and producing an altered protein containing 15 extra amino acids at the C‐terminus [147, 148]. Other TNPO3 mutations have been reported: c.2767delC and c.2757delC, leading to elongated proteins as well [149, 150]; a missense mutation, c.2453G>A, producing a dissimilar phenotype from the typical one [151].

The function of TNPO3 in muscle cells and the relationship between TNPO3 mutations and LGMDD2 clinical features are still not completely understood. The clinical phenotype includes severe muscle weakness, starting in the pelvic girdle muscle and then developing in the shoulder girdle [4, 152]. Sometimes, scoliosis, arachnodactyly, distal muscle and facial weakness, scapular winging, and joint contractures can also be observed in patients [147, 148, 153]. On the opposite, there is no cognitive or cardiac damage, whereas respiratory or bulbar involvement has been reported only in some cases, having early onset and severe phenotype [154]. There is variability both in the onset age, ranging from 1 to 31 years, and in the degree of disability of pelvic girdle muscles [4, 152]. The clinical spectrum, the involved genetic mutations, and the pathogenetic mechanism of LGMDD2 have been reviewed by Costa et al. in 2022 [5].

Although LGMDs have a specific genetic molecular base, a muscle biopsy analysis—by both immunohistochemistry and Western blot—is generally still necessary to perform a correct diagnosis. Nevertheless, this analysis is expensive and invasive, and does not allow the evaluation of the disorder course or the efficacy of a therapeutic treatment. CK levels have often been used as a biomarker, but in some forms of muscular dystrophy they are only slightly increased and affected by the patient's clinical picture and treatment. Hence, the identification of novel non‐invasive biomarkers is highly crucial, to monitor the disorder course and to assess the efficacy of therapeutic options as well. Actually, up to now, LGMD patients have not taken advantage of efficient therapeutic treatments, even though some therapies are under development and various clinical trials are ongoing [132]. That's why miRs seem to be encouraging biomarkers for LGMDs.

## 8 LGMD and myomiRNAs

Many studies demonstrate miRNA involvement in LGMD; therefore, their possible employment as biomarkers has been suggested for diagnosing the pathology, monitoring its development, and evaluating the effect of a possible therapeutic strategy. Two main objectives should be achieved: the first is to distinguish LGMD from other muscular dystrophies, as they all involve progressive muscle wasting and sometimes share the same intracellular pathways and signal molecules; the second is to discriminate between the different forms of LGMD, which have distinct pathogeneses, onset ages, and slightly different clinical traits.

Among the first published works, Matsuzaka et al. in 2014 [60] evaluated the expression of canonical myomiRNAs—miR‐1, miR‐133a/b, and miR‐206—previously found dysregulated in DMD patients. The analysis, performed by RT‐qPCR on serum from patients with various muscular dystrophies, including BMD, DMD, DM1, LGMD, FSHD, and Distal Myopathy with Rimmed Vacuoles (DMRV), suggested that miR‐1, miR‐133a, and miR‐206 could serve as possible biomarkers for BMD, FSHD, and LGMD. However, the results need a larger sample size for more robust conclusions.

More recently, in 2021, Pegoraro and Angelini [4] confirmed that the circulating miR‐206 amount was significantly high in LGMD patients in comparison to healthy subjects. Indeed, they also studied the possible role of the myomiRNA miR‐206 in evaluating the disorder development in patients suffering from LGMD. Eleven patients were enrolled, with distinct forms of LGMD: transportinopathy, sarcoglycanopathy, and calpainopathy. The miR‐206 amount in serum was analyzed by RT‐qPCR, and the values were found significantly higher in LGMD patients than in healthy controls, reaching a peak of expression 50–80 fold higher in two LGMD subjects having a severe and quick disorder progression in transportinopathy and calpainopathy forms. This study provided the first evidence of a possible prognostic use of miR‐206 to monitor disease course.

A more recent work [13] aimed to non‐invasively find a molecular signature for LGMD useful to distinguish LGMD from other muscular dystrophies and for patient prognosis and stratification. Plasmatic miRNAs were analyzed by whole miRNome sequencing in LGMD patients and healthy subjects, comparing them with other muscular dystrophies such as DMD and FSHD. A differential expression of 13 miRNAs was found in LGMD patients compared to controls: 10 of them resulted up‐regulated (miR‐29a‐3p, miR‐122‐5p, miR‐122b‐3p, miR‐192‐5p, miR‐203a‐3p, miR‐203b‐5p, miR‐574‐3p, mir‐885‐3p, miR‐4646‐3p, and miR‐6511a‐3p), while three of them were found significantly down‐regulated (miR‐19b‐3p, miR‐323b‐3p, miR‐7706). After validation by RT‐qPCR, a bioinformatic analysis was performed and target genes were identified, showing that the most affected pathways are muscle development, senescence and regeneration, and cell cycle. In particular, miR‐122‐5p, miR‐192‐5p, and miR‐323b‐3p levels were found to be enough specific and sensitive to discriminate LGMD, DMD, and FSHD patients from healthy subjects, suggesting a possible clinical use for LGMD patients stratification and prognosis.

Furthermore, Mousa et al. [155] studied the diagnostic potential for neuromuscular diseases of some miRNAs involved in physiological and pathological muscle processes: miR‐103a‐3p, miR‐103a‐5p, miR‐191, miR‐206, miR‐208a, miR‐223, miR‐499 [156, 157]. The analysis was performed on peripheral blood samples collected from patients with various forms of muscular dystrophy: BMD, Congenital Muscular Dystrophy (CMD), Congenital Myopathy (CM), DMD, LGMD, and Spinal Muscular Atrophy (SMA), including clinical phenotypes of calpainopathy, dysferlinopathy, sarcoglycanopathy, and titinopathy. By using RT‐qPCR, circulating miR‐499 amount was found to be more elevated in neuromuscular patients compared to controls, with different values in the different diseases, making it possible to distinguish all the tested disorders. On the contrary, in this work, miR‐206 resulted down‐regulated in pathological conditions: it showed a potential to discriminate patients from healthy subjects, to differentiate between DMD and all other diseases but not to distinguish among the other disorders. A significantly down‐regulated level of miR‐208a was found in SMA patients, allowing their discrimination from other neuromuscular patients. Circulating miR‐103a‐3p was down‐regulated in BMD and up‐regulated in DMD, allowing us to distinguish between these disorders but not among other neuromuscular diseases. Instead, miR‐103a‐5p showed a highly significant down‐regulation in 75% of SMA patients in comparison to healthy control subjects and to other diseases. MiR‐191 levels were found low in all patients, and statistical analysis showed that it can distinguish between SMA and other disorders, between DMD and BMD, and between DMD and LGMD. Finally, miR‐223 resulted up‐regulated in all neuromuscular diseases except for SMA, where it was down‐regulated. So it was suggested its possible use to discriminate between SMA and other diseases but not between different muscular dystrophies.

With the same aim as the previous studies, Magri et al. [138] tried to identify the miRNA signature in LGMD for patient screening and stratification. In this study, an array analysis of 179 miRs in serum from 16 LGMD patients and from healthy subjects was performed and subsequently confirmed by RT‐qPCR, resulting in 107 total dysregulated miRs in LGMD patients in comparison to the control group. Among them, six miRNAs appeared down‐regulated: let‐7f‐5p (in LGMDR1), miR‐20a‐5p (in LGMDR2), miR‐130b‐5p and miR‐378a‐5p (both in LGMDR3), miR‐376c‐3p and miR‐382‐5p (both in LGMDR4). The combination of six miRNAs allowed for discrimination of LGMD patients from controls, while the amounts of single miRNAs allowed for identification of a particular LGMD subtype. This is very important since the different LGMD forms have different clinical phenotypes and progression, and the possibility to stratify and manage LGMD patients through specific circulating miRs molecular signatures remains a challenge [13].

Recently, Oliveira et al. [6] published a review about the association between miRNA levels and disease severity, course, and response to therapeutic treatments in animal models of different subtypes of LGMD. Five studies were analyzed, published from 2013 to 2019 [77, 80, 158, 159, 160], evaluating the miRNA expression in muscle, serum, or both of mice models of the following subtypes of LGMD: LGMDR1, −R2, −R3, −R5, and −R6. MiR‐1, miR‐133a, and miR‐206 result differentially expressed in serum and muscle across disease subtypes, with levels depending on muscle regeneration, fibrosis, inflammation degree, and disease course. These miRs are highly expressed in pathological muscles, and reversed dose‐dependently after treatment, confirming their involvement in disease progression, seriousness, therapeutic response, and suitability as clinical biomarkers.

Finally, Aguennouz et al. in 2016 [127] carried out a study about skeletal muscle miRNA expression in three cohorts of patients with different forms of calpainopathy: primary deficiency (G1), autocatalytic activity deficiency (G2), and secondary deficiency (G3) or dysferlinopathy, respectively. Healthy subjects were used as controls. The analysis was performed by TLDA. Among the 384 analyzed miRs, 187 total miRNAs resulted significantly dysregulated in all patients in comparison to controls. In particular, 17 miRNAs displayed a similar expression trend in all the analyzed cohorts. For example, 5 miRNAs, involved in cell growth and differentiation, were up‐regulated: miR‐34a and miR‐198, reducing motogenic and mitogenic pathways and thus decreasing cell growth and movement; miR‐432, with a role in muscle development; miR‐503, promoting cell cycle arrest; and miR‐650, involved in cell proliferation and survival. In addition, other miRNAs resulted differentially expressed in the three analyzed patient cohorts, suggesting that the onset and course of the specific diseases involve different molecular mechanisms and could serve as potential biomarkers. In particular, 29 miRNAs were dysregulated only in G1 patients, 45 miRNAs only in G2 patients, and 14 miRNAs only in G3 patients, whereas 6 miRNAs had an opposite regulation in G1 and G2 versus G3: miR‐154, miR‐184, miR‐485‐3p, miR‐493, miR‐519e, and miR‐654. The last two miRNAs have a role in cell‐cycle arrest, an important process for muscle regeneration. MiR‐200, which was found more expressed in primary CAPN3 deficit than in CAPN3 autocatalysis deficit, is involved in myoblast fusion as a compensatory way in the dystrophic muscle to promote muscle regeneration. In the secondary deficit, two miRNAs resulted strongly down‐regulated, miR‐184 and miR‐493, both having as a target the calcium metabolic pathway and being involved in inflammatory processes as well. As previously reported, also miR‐143‐3p and miR‐486‐3p serum levels were found dysregulated in LGMDR1 patients in comparison to control subjects [63].

Overall, these studies underline the possible use of miRs as diagnostic biomarkers for LGMD, as well as to stratify patients and evaluate therapeutic strategies. However, further research and validation in larger patient groups are necessary to confirm their potential clinical application.

## 9 Conclusion and Perspectives

Muscular dystrophies, including LGMD, are rare and heterogeneous neuromuscular disorders showing increasing muscle wasting and degeneration. For these diseases, a definite therapy is not available yet, even though several approaches are under development [73, 161]. An early specific diagnosis remains crucial for both genetic counseling and effective clinical management of patients [5].

Currently, CK levels in serum are regularly employed as a clinical biomarker to diagnose muscular dystrophies since they are generally increased in patients [64]. However, CK values are dysregulated in a variety of conditions, such as vigorous exercise, decreased renal function, pregnancy, alcohol consumption, and skeletal muscle mass [162]. In combination with CK levels and clinical examination, a muscle biopsy is generally needed to discriminate among different forms of muscular dystrophy, but it is invasive and expensive, and it does not allow for evaluating the disorder progression or for assessing the effects of a therapeutic intervention. Genetic tests can also help with the diagnoses, whereas physical examinations of patients by the clinicians remain the main tool to follow the progress of the disease.

That's why new non‐invasive biomarkers are highly needed. Indeed, in recent years the interest in biomarkers identification for this class of disorders is progressively increasing. Nevertheless, the greatest effort was made in identifying biomarkers for DMD, whereas a lower number of studies have been reported to address the rarer forms of muscular dystrophies, including LGMD.

Even if LGMD is a rare disease with variable progression, some clinical symptoms can be handled. Therefore, in addition to the other previously mentioned reasons, it should be useful to be able to discover patients susceptible to a quicker disease development. Non‐invasive and accurate biomarkers are required to give information about diagnosis, clinical parameters, and progression rate.

MiRs are good biomarker candidates for neuromuscular diseases, including muscular dystrophies, as they remain stable in blood and participate in the regulation of muscular protein expression, in the maintenance of healthy muscle status, and in muscle cell regeneration. In addition, they have been found dysregulated in pathological conditions, and their levels are often related to disease severity and progression. A lot of studies were performed in this respect, and the possible employment of miRNAs as biomarkers has already been described for various disorders and conditions [20].

Many miRNAs have been identified as potentially useful to discriminate muscular dystrophy patients from healthy individuals because of a different expression, and their number is continuously increasing thanks to the availability of high‐throughput detection techniques.

In particular, myomiRNA detection in skeletal muscle is primarily achieved through biopsy, followed by homogenization and RNA extraction using methods like phenol‐chloroform or commercial kits to yield a high concentration of RNA [163]. These muscle‐specific miRNAs are then quantified using highly sensitive and specific techniques such as qRT‐PCR, which is considered the gold standard, or high‐throughput methods like NGS and microarrays [164]. In contrast, serum myomiRNA detection is a non‐invasive approach that faces the challenge of low RNA concentration and the presence of inhibitors. It requires specialized extraction kits and can be prone to contamination from hemolysis [81]. Due to the limited amount of myomiRNAs, quantification often necessitates highly sensitive methods like pre‐amplification before qRT‐PCR or the use of Digital PCR (dPCR) for absolute quantification [165]. While skeletal muscle provides a rich source for analyzing myomiRNA expression, serum offers a powerful tool for biomarker discovery and disease monitoring. Both techniques require rigorous standardization to ensure reliable and reproducible results, with a key challenge in both tissues being the selection of appropriate internal controls for accurate normalization.

Regarding the specific myomiRNAs, some of them, as the canonical myomiRNAs miR‐206, miR‐1, and miR‐133, are shared by different muscular dystrophies, whereas others seem to be specific to the type of muscular dystrophy, as already reported [3, 63, 72, 76]. Nevertheless, it is still difficult to identify the most relevant altered myomiRNAs that could be promising for diagnosis or prognosis of each disease. Indeed, published studies are very heterogeneous for the number of investigated miRNAs and the type of analyzed biological material (muscle biopsy or isolated cells, plasma or serum), since also a quantitative difference between circulating miRNAs and miRNA levels in muscle tissue should be expected. In addition, they enroll a few number of available patients, different from one study to another and having different clinical parameters and characteristics. Therefore, the results obtained by the different studies described in literature are often uncomparable. Moreover, further analyses of dysregulated miRNAs are required to investigate their target genes, their biological role, and the pathways they are involved in for each type of considered muscular dystrophy.

In LGMDs, circulating miR levels could be helpful as non‐invasive molecular biomarkers both to specifically identify and evaluate LGMD patients, and to discriminate the different LGMD subtypes. They can give a kind of molecular signature useful as a reference point for patient stratification and management, for disease monitoring, and to evaluate the effects of a therapeutic approach [10].

Among the different published and previously described studies, a lot of miRNAs, including the universally recognized myomiRNAs, were analyzed, and several of them were found differentially expressed in LGMD patients with respect to other neuromuscular patients. In addition, different miR levels were found among the different LGMD subtypes. Such works have additionally increased our understanding of LGMD pathophysiology, and some identified miRs have been proposed as possible biomarkers for LGMD.

Nevertheless, some issues should be addressed: first, neuromuscular diseases are rare, limiting the number of samples available to identify a specific molecular signature, and therefore requiring further validation of the experimental results in a higher number of patients. Second, as a consequence, some studies obtained opposite results regarding the expression of the same miRNA, or the results of different studies were not overlapping. A lot of potentially useful miRNAs were identified, but sometimes neither their molecular targets nor the cellular pathways they are involved in are known. As also underlined by Mousa et al. in 2023 [155], it would be helpful to clarify the exact miRNAs' role in muscle differentiation and regeneration to better understand their roles in different muscle disorders.

In conclusion, while various miRNAs have been suggested as possible biomarkers for LGMD, currently no validated miRNA biomarkers have been described for clinical use yet. Further research is necessary to better understand both physiological and pathological processes in muscle cells, and to evaluate the reliability of these miRNAs as biomarkers for LGMD diagnosis, progression, and therapeutic monitoring.

## Conflicts of Interest

The authors declare no conflicts of interest.

## References

[1] J. S. Mattick and I. V. Makunin , “Non‐Coding RNA,” Human Molecular Genetics 15 (2006): R17–R29. 16651366 10.1093/hmg/ddl046

[2] T. J. Kirby , T. Chaillou , and J. J. McCarthy , “The Role of microRNAs in Skeletal Muscle Health and Disease,” Frontiers in Bioscience 20 (2015): 37–77. 10.2741/4298 PMC4853752 25553440

[3] I. Eisenberg , A. Eran , I. Nishino , et al., “Distinctive Patterns of microRNA Expression in Primary Muscular Disorders,” Proceedings of the National Academy of Sciences of the United States of America 104 (2007): 17016–17021. 17942673 10.1073/pnas.0708115104 PMC2040449

[4] V. Pegoraro and C. Angelini , “Circulating miR‐206 as a Biomarker for Patients Affected by Severe Limb Girdle Muscle Dystrophies,” Genes 12 (2021): 85. 33445560 10.3390/genes12010085 PMC7826967

[5] R. Costa , M. T. Rodia , S. Pacilio , C. Angelini , and G. Cenacchi , “LGMD D2 TNPO3‐Related: From Clinical Spectrum to Pathogenetic Mechanism,” Frontiers in Neurology 13 (2022): 840683. 35309568 10.3389/fneur.2022.840683 PMC8931187

[6] M. T. J. S. Oliveira , T. A. B. da Silva Santana , M. C. M. Costa , et al., “MicroRNA as Potential Biomarker for Severity, Progression, and Therapeutic Monitoring in Animal Models of Limb‐Girdle Muscular Dystrophy: A Systematic Review,” Frontiers in Cellular Neuroscience 17 (2023): 1233181. 38130868 10.3389/fncel.2023.1233181 PMC10733523

[7] R. C. Lee , R. L. Feinbaum , and V. Ambros , “The *C. elegans* Heterochronic Gene Lin‐4 Encodes Small RNAs With Antisense Complementarity to Lin‐14,” Cell 75 (1993): 843–854. 8252621 10.1016/0092-8674(93)90529-y

[8] S. Y. Ying , D. C. Chang , and S. L. Lin , “The MicroRNA,” Methods in Molecular Biology 1733 (2018): 1–25. 29435919 10.1007/978-1-4939-7601-0_1

[9] L. L. Chen and V. N. Kim , “Small and Long Non‐Coding RNAs: Past, Present, and Future,” Cell 187 (2024): 6451–6485. 39547208 10.1016/j.cell.2024.10.024

[10] M. S. Alexander and L. M. Kunkel , “Skeletal Muscle MicroRNAs: Their Diagnostic and Therapeutic Potential in Human Muscle Diseases,” Journal of Neuromuscular Diseases 2 (2015): 1–11. 27547731 10.3233/JND-140058 PMC4988517

[11] G. F. Mok , E. Lozano‐Velasco , and A. Münsterberg , “microRNAs in Skeletal Muscle Development,” Seminars in Cell and Developmental Biology 72 (2017): 67–76. 29102719 10.1016/j.semcdb.2017.10.032

[12] I. Monga and M. Kumar , “Computational Resources for Prediction and Analysis of Functional miRNA and Their Targetome,” Methods in Molecular Biology 1912 (2019): 215–250. 30635896 10.1007/978-1-4939-8982-9_9

[13] J. L. García‐Giménez , E. R. García‐Trevijano , A. I. Avilés‐Alía , et al., “Identification of Circulating miRNAs Differentially Expressed in Patients With Limb‐Girdle, Duchenne or Facioscapulohumeral Muscular Dystrophies,” Orphanet Journal of Rare Diseases 17 (2022): 450. 36575500 10.1186/s13023-022-02603-3 PMC9793535

[14] D. P. Bartel , “MicroRNAs: Genomics, Biogenesis, Mechanism, and Function,” Cell 116 (2004): 281–297. 14744438 10.1016/s0092-8674(04)00045-5

[15] S. Srivastava , R. Rathor , S. N. Singh , and G. Suryakumar , “Emerging Role of MyomiRs as Biomarkers and Therapeutic Targets in Skeletal Muscle Diseases,” American Journal of Physiology. Cell Physiology 321 (2021): C859–C875. 34586896 10.1152/ajpcell.00057.2021

[16] Z. Latifi , S. Nikanfar , R. Khodavirdilou , et al., “MicroRNAs as Diagnostic Biomarkers in Diabetes Male Infertility: A Systematic Review,” Molecular Biology Reports 52 (2024): 90. 39739064 10.1007/s11033-024-10197-1

[17] E. Podyacheva , J. Snezhkova , A. Onopchenko , V. Dyachuk , and Y. Toropova , “The Role of MicroRNAs in the Pathogenesis of Doxorubicin‐Induced Vascular Remodeling,” International Journal of Molecular Sciences 25 (2024): 13335. 39769102 10.3390/ijms252413335 PMC11728060

[18] S. V. Kapplingattu , S. Bhattacharya , and Y. K. Adlakha , “MiRNAs as Major Players in Brain Health and Disease: Current Knowledge and Future Perspectives,” Cell Death Discovery 11 (2025): 7. 39805813 10.1038/s41420-024-02283-x PMC11729916

[19] S. Gilad , E. Meiri , Y. Yogev , et al., “Serum microRNAs Are Promising Novel Biomarkers,” PLoS One 3 (2008): e3148. 18773077 10.1371/journal.pone.0003148 PMC2519789

[20] W. Huang , “MicroRNAs: Biomarkers, Diagnostics, and Therapeutics,” Methods in Molecular Biology 1617 (2017): 57–67. 28540676 10.1007/978-1-4939-7046-9_4

[21] A. Koutsoulidou and L. A. Phylactou , “Circulating Biomarkers in Muscular Dystrophies: Disease and Therapy Monitoring,” Molecular Therapy ‐ Methods & Clinical Development 18 (2020): 230–239. 32637452 10.1016/j.omtm.2020.05.017 PMC7327849

[22] M. Sharma , P. K. Juvvuna , H. Kukreti , and C. McFarlane , “Mega Roles of microRNAs in Regulation of Skeletal Muscle Health and Disease,” Frontiers in Physiology 5 (2014): 239. 25018733 10.3389/fphys.2014.00239 PMC4072100

[23] M. O. Ott , E. Bober , G. Lyons , H. Arnold , and M. Buckingham , “Early Expression of the Myogenic Regulatory Gene, Myf‐5, in Precursor Cells of Skeletal Muscle in the Mouse Embryo,” Development 111 (1991): 1097–1107. 1652425 10.1242/dev.111.4.1097

[24] D. Sassoon , G. Lyons , W. E. Wright , et al., “Expression of Two Myogenic Regulatory Factors Myogenin and MyoD1 During Mouse Embryogenesis,” Nature 341 (1989): 303–307. 2552320 10.1038/341303a0

[25] E. Bober , G. E. Lyons , T. Braun , et al., “The Muscle Regulatory Gene, Myf‐6, Has a Biphasic Pattern of Expression During Early Mouse Development,” Journal of Cell Biology 113 (1991): 1255–1265. 2045411 10.1083/jcb.113.6.1255 PMC2289041

[26] T. J. Hinterberger , D. A. Sassoon , S. J. Rhodes , and S. F. Konieczny , “Expression of the Muscle Regulatory Factor MRF4 During Somite and Skeletal Myofiber Development,” Developmental Biology 147 (1991): 144–156. 1715299 10.1016/s0012-1606(05)80014-4

[27] M. Ballarino , M. Morlando , A. Fatica , and I. Bozzoni , “Non‐Coding RNAs in Muscle Differentiation and Musculoskeletal Disease,” Journal of Clinical Investigation 126 (2016): 2021–2030. 27249675 10.1172/JCI84419 PMC4887180

[28] M. Horak , J. Novak , and J. Bienertova‐Vasku , “Muscle‐Specific microRNAs in Skeletal Muscle Development,” Developmental Biology 410 (2016): 1–13. 26708096 10.1016/j.ydbio.2015.12.013

[29] W. H. Townley‐Tilson , T. E. Callis , and D. Wang , “MicroRNAs 1, 133, and 206: Critical Factors of Skeletal and Cardiac Muscle Development, Function, and Disease,” International Journal of Biochemistry and Cell Biology 42 (2010): 1252–1255. 20619221 10.1016/j.biocel.2009.03.002 PMC2904322

[30] J. F. Chen , E. M. Mandel , J. M. Thomson , et al., “The Role of microRNA‐1 and microRNA‐133 in Skeletal Muscle Proliferation and Differentiation,” Nature Genetics 38 (2006): 228–233. 16380711 10.1038/ng1725 PMC2538576

[31] J. J. McCarthy , “MicroRNA‐206: The Skeletal Muscle‐Specific myomiR,” Biochimica et Biophysica Acta 1779 (2008): 682–691. 18381085 10.1016/j.bbagrm.2008.03.001 PMC2656394

[32] A. Koutsoulidou , N. P. Mastroyiannopoulos , D. Furling , J. B. Uney , and L. A. Phylactou , “Expression of miR‐1, miR‐133a, miR‐133b and miR‐206 Increases During Development of Human Skeletal Muscle,” BMC Developmental Biology 11 (2011): 34. 21645416 10.1186/1471-213X-11-34 PMC3132729

[33] K. R. Mitchelson and W. Y. Qin , “Roles of the Canonical myomiRs miR‐1, −133 and −206 in Cell Development and Disease,” World Journal of Biological Chemistry 6 (2015): 162–208. 26322174 10.4331/wjbc.v6.i3.162 PMC4549760

[34] E. M. Small , J. R. O'Rourke , V. Moresi , et al., “Regulation of PI3‐Kinase/Akt Signaling by Muscle‐Enriched microRNA‐486,” Proceedings of the National Academy of Sciences of the United States of America 107 (2010): 4218–4223. 20142475 10.1073/pnas.1000300107 PMC2840099

[35] J. J. McCarthy and K. A. Esser , “MicroRNA‐1 and microRNA‐133a Expression Are Decreased During Skeletal Muscle Hypertrophy,” Journal of Applied Physiology 102 (2007): 306–313. 17008435 10.1152/japplphysiol.00932.2006

[36] T. E. Callis , Z. Deng , J. F. Chen , and D. Z. Wang , “Muscling Through the microRNA World,” Experimental Biology and Medicine 233 (2008): 131–138. 18222968 10.3181/0709-MR-237

[37] T. B. Walden , J. A. Timmons , P. Keller , J. Nedergaard , and B. Cannon , “Distinct Expression of Muscle‐Specific microRNAs (Myomirs) in Brown Adipocytes,” Journal of Cellular Physiology 218 (2009): 444–449. 18937285 10.1002/jcp.21621

[38] Y. Ge and J. Chen , “MicroRNAs in Skeletal Myogenesis,” Cell Cycle 10 (2011): 441–448. 21270519 10.4161/cc.10.3.14710 PMC3115018

[39] N. Nohata , T. Hanazawa , H. Enokida , and N. Seki , “microRNA‐1/133a and microRNA‐206/133b Clusters: Dysregulation and Functional Roles in Human Cancers,” Oncotarget 3 (2012): 9–21. 22308266 10.18632/oncotarget.424 PMC3292888

[40] E. van Rooij , D. Quiat , B. A. Johnson , et al., “A Family of microRNAs Encoded by Myosin Genes Governs Myosin Expression and Muscle Performance,” Developmental Cell 17 (2009): 662–673. 19922871 10.1016/j.devcel.2009.10.013 PMC2796371

[41] G. P. Diniz and D. Z. Wang , “Regulation of Skeletal Muscle by microRNAs,” Comprehensive Physiology 6 (2016): 1279–1294. 27347893 10.1002/cphy.c150041

[42] T. J. Kirby and J. J. McCarthy , “MicroRNAs in Skeletal Muscle Biology and Exercise Adaptation,” Free Radical Biology and Medicine 64 (2013): 95–105. 23872025 10.1016/j.freeradbiomed.2013.07.004 PMC4867469

[43] A. Kovanda , T. Režen , and B. Rogelj , “MicroRNA in Skeletal Muscle Development, Growth, Atrophy, and Disease,” Wiley Interdisciplinary Reviews: RNA 5 (2014): 509–525. 24838768 10.1002/wrna.1227

[44] J. Wang , L. Z. Yang , J. S. Zhang , et al., “Effects of microRNAs on Skeletal Muscle Development,” Gene 668 (2018): 107–113. 29775754 10.1016/j.gene.2018.05.039

[45] H. K. Kim , Y. S. Lee , U. Sivaprasad , A. Malhotra , and A. Dutta , “Muscle‐Specific microRNA miR‐206 Promotes Muscle Differentiation,” Journal of Cell Biology 174 (2006): 677–687. 16923828 10.1083/jcb.200603008 PMC2064311

[46] K. Goljanek‐Whysall , H. Pais , T. Rathjen , D. Sweetman , T. Dalmay , and A. Münsterberg , “Regulation of Multiple Target Genes by miR‐1 and miR‐206 Is Pivotal for C2C12 Myoblast Differentiation,” Journal of Cell Science 125 (2012): 3590–3600. 22595520 10.1242/jcs.101758

[47] A. H. Williams , N. Liu , E. van Rooij , and E. N. Olson , “MicroRNA Control of Muscle Development and Disease,” Current Opinion in Cell Biology 21 (2009): 461–469. 19278845 10.1016/j.ceb.2009.01.029 PMC2692369

[48] P. K. Rao , R. M. Kumar , M. Farkhondeh , S. Baskerville , and H. F. Lodish , “Myogenic Factors That Regulate Expression of Muscle‐Specific microRNAs,” Proceedings of the National Academy of Sciences of the United States of America 103 (2006): 8721–8726. 16731620 10.1073/pnas.0602831103 PMC1482645

[49] M. I. Rosenberg , S. A. Georges , A. Asawachaicharn , E. Analau , and S. J. Tapscott , “MyoD Inhibits Fstl1 and Utrn Expression by Inducing Transcription of miR‐206,” Journal of Cell Biology 175 (2006): 77–85. 17030984 10.1083/jcb.200603039 PMC2064500

[50] D. Sweetman , K. Goljanek , T. Rathjen , et al., “Specific Requirements of MRFs for the Expression of Muscle Specific microRNAs, miR‐1, miR‐206 and miR‐133,” Developmental Biology 321 (2008): 491–499. 18619954 10.1016/j.ydbio.2008.06.019

[51] C. F. Wong and R. L. Tellam , “MicroRNA‐26a Targets the Histone Methyltransferase Enhancer of Zeste Homolog 2 During Myogenesis,” Journal of Biological Chemistry 283 (2008): 9836–9843. 18281287 10.1074/jbc.M709614200

[52] C. G. Crist , D. Montarras , G. Pallafacchina , et al., “Muscle Stem Cell Behavior Is Modified by microRNA‐27 Regulation of Pax3 Expression,” Proceedings of the National Academy of Sciences of the United States of America 106 (2009): 13383–13387. 19666532 10.1073/pnas.0900210106 PMC2726381

[53] I. Naguibneva , M. Ameyar‐Zazoua , A. Polesskaya , et al., “The microRNA miR‐181 Targets the Homeobox Protein Hox‐A11 During Mammalian Myoblast Differentiation,” Nature Cell Biology 8 (2006): 278–284. 16489342 10.1038/ncb1373

[54] B. Cardinali , L. Castellani , P. Fasanaro , et al., “Microrna‐221 and Microrna‐222 Modulate Differentiation and Maturation of Skeletal Muscle Cells,” PLoS One 4 (2009): e7607. 19859555 10.1371/journal.pone.0007607 PMC2762614

[55] B. Liu , Y. Shi , H. He , et al., “miR‐221 Modulates Skeletal Muscle Satellite Cells Proliferation and Differentiation,” In Vitro Cellular and Developmental Biology–Animal 54 (2018): 147–155. 29197032 10.1007/s11626-017-0210-x

[56] S. Sarkar , B. K. Dey , and A. Dutta , “MiR‐322/424 and −503 Are Induced During Muscle Differentiation and Promote Cell Cycle Quiescence and Differentiation by Down‐Regulation of Cdc25A,” Molecular Biology of the Cell 21 (2010): 2138–2149. 20462953 10.1091/mbc.E10-01-0062 PMC2893979

[57] D. Castel , M. B. Baghdadi , S. Mella , et al., “Small‐RNA Sequencing Identifies Dynamic microRNA Deregulation During Skeletal Muscle Lineage Progression,” Scientific Reports 8 (2018): 4208. 29523801 10.1038/s41598-018-21991-w PMC5844870

[58] J. Novák , J. Vinklárek , J. Bienertová‐Vašků , and O. Slabý , “MicroRNAs Involved in Skeletal Muscle Development and Their Roles in Rhabdomyosarcoma Pathogenesis,” Pediatric Blood and Cancer 60 (2013): 1739–1746. 23813576 10.1002/pbc.24664

[59] R. W. Georgantas , K. Streicher , S. A. Greenberg , et al., “Inhibition of Myogenic microRNAs 1, 133, and 206 by Inflammatory Cytokines Links Inflammation and Muscle Degeneration in Adult Inflammatory Myopathies,” Arthritis & Rhematology 66 (2014): 1022–1033. 10.1002/art.38292 24757153

[60] Y. Matsuzaka , S. Kishi , Y. Aoki , et al., “Three Novel Serum Biomarkers, miR‐1, miR‐133a, and miR‐206 for Limb‐Girdle Muscular Dystrophy, Facioscapulohumeral Muscular Dystrophy, and Becker Muscular Dystrophy,” Environmental Health and Preventive Medicine 19 (2014): 452–458. 25150707 10.1007/s12199-014-0405-7 PMC4235845

[61] K. Arahata , “Muscular Dystrophy,” Neuropathology 20 (2000): S34–S41. 11037185 10.1046/j.1440-1789.2000.00321.x

[62] A. E. Emery , “The Muscular Dystrophies,” Lancet 359 (2002): 687–695. 11879882 10.1016/S0140-6736(02)07815-7

[63] A. Koutsoulidou , D. Koutalianos , K. Georgiou , et al., “Serum miRNAs as Biomarkers for the Rare Types of Muscular Dystrophy,” Neuromuscular Disorders 32 (2022): 332–346. 35393236 10.1016/j.nmd.2022.03.003

[64] I. Dabaj , F. Ducatez , S. Marret , S. Bekri , and A. Tebani , “Neuromuscular Disorders in the Omics Era,” Clinica Chimica Acta 553 (2024): 117691. 10.1016/j.cca.2023.117691 38081447

[65] E. Mercuri and F. Muntoni , “Muscular Dystrophies,” Lancet 381 (2013): 845–860. 23465426 10.1016/S0140-6736(12)61897-2

[66] E. Cohen , G. Bonne , F. Rivier , and D. Hamroun , “The 2022 Version of the Gene Table of Neuromuscular Disorders (Nuclear Genome),” Neuromuscular Disorders 31 (2021): 1313–1357. 34930546 10.1016/j.nmd.2021.11.004

[67] D. Duan , N. Goemans , S. Takeda , E. Mercuri , and A. Aartsma‐Rus , “Duchenne Muscular Dystrophy,” Nature Reviews. Disease Primers 7 (2021): 13. 10.1038/s41572-021-00248-3 PMC10557455 33602943

[68] V. Straub and M. Guglieri , “An Update on Becker Muscular Dystrophy,” Current Opinion in Neurology 36 (2023): 450–454. 37591308 10.1097/WCO.0000000000001191 PMC10487383

[69] V. Salsi , G. N. A. Vattemi , and R. G. Tupler , “The FSHD Jigsaw: Are We Placing the Tiles in the Right Position?,” Current Opinion in Neurology 36 (2023): 455–463. 37338810 10.1097/WCO.0000000000001176 PMC10487374

[70] S. LoRusso , B. Weiner , and W. D. Arnold , “Myotonic Dystrophies: Targeting Therapies for Multisystem Disease,” Neurotherapeutics 15 (2018): 872–884. 30341596 10.1007/s13311-018-00679-z PMC6277298

[71] N. E. Johnson and J. M. Statland , “The Limb‐Girdle Muscular Dystrophies,” Continuum 28 (2022): 1698–1714. 36537976 10.1212/CON.0000000000001178

[72] A. C. Kakouri , D. Koutalianos , A. Koutsoulidou , et al., “Circulating Small RNA Signatures Differentiate Accurately the Subtypes of Muscular Dystrophies: Small‐RNA Next‐Generation Sequencing Analytics and Functional Insights,” RNA Biology 19 (2022): 507–518. 35388741 10.1080/15476286.2022.2058817 PMC8993092

[73] Y. Chulanova , D. Breier , and D. Peer , “Delivery of Genetic Medicines for Muscular Dystrophies,” Cell Reports Medicine 6 (2025): 101885. 39765231 10.1016/j.xcrm.2024.101885 PMC11866442

[74] E. Zacharewicz , S. Lamon , and A. P. Russell , “MicroRNAs in Skeletal Muscle and Their Regulation With Exercise, Ageing, and Disease,” Frontiers in Physiology 4 (2013): 266. 24137130 10.3389/fphys.2013.00266 PMC3786223

[75] D. Cacchiarelli , I. Legnini , J. Martone , et al., “miRNAs as Serum Biomarkers for Duchenne Muscular Dystrophy,” EMBO Molecular Medicine 3 (2011): 258–265. 21425469 10.1002/emmm.201100133 PMC3112257

[76] K. Kiełbowski , E. Bakinowska , G. Procyk , M. Ziętara , and A. Pawlik , “The Role of MicroRNA in the Pathogenesis of Duchenne Muscular Dystrophy,” International Journal of Molecular Sciences 25 (2024): 6108. 38892293 10.3390/ijms25116108 PMC11172814

[77] D. Israeli , J. Poupiot , F. Amor , et al., “Circulating miRNAs Are Generic and Versatile Therapeutic Monitoring Biomarkers in Muscular Dystrophies,” Scientific Reports 6 (2016): 28097. 27323895 10.1038/srep28097 PMC4914855

[78] X. Li , Y. Li , L. Zhao , et al., “Circulating Muscle‐Specific miRNAs in Duchenne Muscular Dystrophy Patients,” Molecular Therapy–Nucleic Acids 3 (2014): e177. 25050825 10.1038/mtna.2014.29 PMC4121518

[79] J. Hu , M. Kong , Y. Ye , S. Hong , L. Cheng , and L. Jiang , “Serum miR‐206 and Other Muscle‐Specific microRNAs as Non‐Invasive Biomarkers for Duchenne Muscular Dystrophy,” Journal of Neurochemistry 129 (2014): 877–883. 24460924 10.1111/jnc.12662

[80] N. Vignier , F. Amor , P. Fogel , et al., “Distinctive Serum miRNA Profile in Mouse Models of Striated Muscular Pathologies,” PLoS One 8 (2013): e55281. 23418438 10.1371/journal.pone.0055281 PMC3572119

[81] H. Mizuno , A. Nakamura , Y. Aoki , et al., “Identification of Muscle‐Specific microRNAs in Serum of Muscular Dystrophy Animal Models: Promising Novel Blood‐Based Markers for Muscular Dystrophy,” PLoS One 6 (2011): e18388. 21479190 10.1371/journal.pone.0018388 PMC3068182

[82] T. C. Roberts , K. E. Blomberg , and G. McClorey , “Expression Analysis in Multiple Muscle Groups and Serum Reveals Complexity in the microRNA Transcriptome of the mdx Mouse With Implications for Therapy,” Molecular Therapy–Nucleic Acids 1 (2012): e39. 23344181 10.1038/mtna.2012.26 PMC3437806

[83] L. Jeanson‐Leh , J. Lameth , S. Krimi , et al., “Serum Profiling Identifies Novel Muscle miRNA and Cardiomyopathy‐Related miRNA Biomarkers in Golden Retriever Muscular Dystrophy Dogs and Duchenne Muscular Dystrophy Patients,” American Journal of Pathology 184 (2014): 2885–2898. 25194663 10.1016/j.ajpath.2014.07.021

[84] C. Colussi , C. Mozzetta , A. Gurtner , et al., “HDAC2 Blockade by Nitric Oxide and Histone Deacetylase Inhibitors Reveals a Common Target in Duchenne Muscular Dystrophy Treatment,” Proceedings of the National Academy of Sciences of the United States of America 105 (2008): 19183–19187. 19047631 10.1073/pnas.0805514105 PMC2614736

[85] D. Cacchiarelli , J. Martone , E. Girardi , et al., “MicroRNAs Involved in Molecular Circuitries Relevant for the Duchenne Muscular Dystrophy Pathogenesis Are Controlled by the Dystrophin/nNOS Pathway,” Cell Metabolism 12 (2010): 341–351. 20727829 10.1016/j.cmet.2010.07.008

[86] N. Liu , A. H. Williams , J. M. Maxeiner , et al., “microRNA‐206 Promotes Skeletal Muscle Regeneration and Delays Progression of Duchenne Muscular Dystrophy in Mice,” Journal of Clinical Investigation 122 (2012): 2054–2065. 22546853 10.1172/JCI62656 PMC3366415

[87] D. Cacchiarelli , T. Incitti , J. Martone , et al., “miR‐31 Modulates Dystrophin Expression: New Implications for Duchenne Muscular Dystrophy Therapy,” EMBO Reports 12 (2011): 136–141. 21212803 10.1038/embor.2010.208 PMC3049433

[88] R. Perbellini , S. Greco , G. Sarra‐Ferraris , et al., “Dysregulation and Cellular Mislocalization of Specific miRNAs in Myotonic Dystrophy Type 1,” Neuromuscular Disorders 21 (2011): 81–88. 21169019 10.1016/j.nmd.2010.11.012

[89] S. Greco , A. Perfetti , P. Fasanaro , et al., “Deregulated microRNAs in Myotonic Dystrophy Type 2,” PLoS One 7 (2012): e39732. 22768114 10.1371/journal.pone.0039732 PMC3387258

[90] A. Koutsoulidou , T. C. Kyriakides , G. K. Papadimas , et al., “Elevated Muscle‐Specific miRNAs in Serum of Myotonic Dystrophy Patients Relate to Muscle Disease Progress,” PLoS One 10 (2015): e0125341. 25915631 10.1371/journal.pone.0125341 PMC4411125

[91] A. Perfetti , S. Greco , R. Cardani , et al., “Validation of Plasma microRNAs as Biomarkers for Myotonic Dystrophy Type 1,” Scientific Reports 6 (2016): 38174. 27905532 10.1038/srep38174 PMC5131283

[92] N. Harafuji , P. Schneiderat , M. C. Walter , and Y. W. Chen , “miR‐411 Is Up‐Regulated in FSHD Myoblasts and Suppresses Myogenic Factors,” Orphanet Journal of Rare Diseases 8 (2013): 55. 23561550 10.1186/1750-1172-8-55 PMC3637251

[93] P. Dmitriev , L. Stankevicins , E. Ansseau , et al., “Defective Regulation of microRNA Target Genes in Myoblasts From Facioscapulohumeral Dystrophy Patients,” Journal of Biological Chemistry 288 (2013): 34989–35002. 24145033 10.1074/jbc.M113.504522 PMC3853252

[94] A. J. Keane , C. Sanz‐Nogués , D. Jayasooriya , et al., “miR‐1, miR‐133a, miR‐29b and Skeletal Muscle Fibrosis in Chronic Limb‐Threatening Ischaemia,” Scientific Reports 14 (2024): 29393. 39592654 10.1038/s41598-024-76415-9 PMC11599917

[95] S. Zanotti , S. Gibertini , M. Curcio , et al., “Opposing Roles of miR‐21 and miR‐29 in the Progression of Fibrosis in Duchenne Muscular Dystrophy,” Biochimica et Biophysica Acta 1852 (2015): 1451–1464. 25892183 10.1016/j.bbadis.2015.04.013

[96] Y. Sun , Y. Li , H. Wang , et al., “miR‐146a‐5p Acts as a Negative Regulator of TGF‐β Signaling in Skeletal Muscle After Acute Contusion,” Acta Biochimica et Biophysica Sinica 49 (2017): 628–634. 28510617 10.1093/abbs/gmx052

[97] I. Bronisz‐Budzyńska , K. Chwalenia , O. Mucha , et al., “miR‐146a Deficiency Does Not Aggravate Muscular Dystrophy in mdx Mice,” Skeletal Muscle 9 (2019): 22. 31412923 10.1186/s13395-019-0207-0 PMC6693262

[98] H. Yao , J. Qian , X. T. Bian , L. Guo , K. L. Tang , and X. Tao , “miR‐27b‐3p Reduces Muscle Fibrosis During Chronic Skeletal Muscle Injury by Targeting TGF‐βR1/Smad Pathway,” Journal of Orthopaedic Surgery and Research 19 (2024): 329. 38825706 10.1186/s13018-024-04733-9 PMC11145862

[99] S. Zanotti , S. Gibertini , F. Blasevich , et al., “Exosomes and Exosomal miRNAs From Muscle‐Derived Fibroblasts Promote Skeletal Muscle Fibrosis,” Matrix Biology 74 (2018): 77–100. 29981373 10.1016/j.matbio.2018.07.003

[100] Y. Sun , H. Wang , Y. Li , S. Liu , J. Chen , and H. Ying , “miR‐24 and miR‐122 Negatively Regulate the Transforming Growth Factor‐β/Smad Signaling Pathway in Skeletal Muscle Fibrosis,” Molecular Therapy–Nucleic Acids 11 (2018): 528–537. 29858088 10.1016/j.omtn.2018.04.005 PMC5992481

[101] X. Song , F. Liu , M. Chen , et al., “MiR‐21 Regulates Skeletal Muscle Atrophy and Fibrosis by Targeting TGF‐Beta/SMAD7‐SMAD2/3 Signaling Pathway,” Heliyon 10 (2024): e33062. 39027432 10.1016/j.heliyon.2024.e33062 PMC11254527

[102] M. Fukuoka , H. Fujita , K. Numao , et al., “MiR‐199‐3p Enhances Muscle Regeneration and Ameliorates Aged Muscle and Muscular Dystrophy,” Communications Biology 4 (2021): 427. 33782502 10.1038/s42003-021-01952-2 PMC8007565

[103] B. K. Dey , K. Pfeifer , and A. Dutta , “The H19 Long Noncoding RNA Gives Rise to microRNAs miR‐675‐3p and miR‐675‐5p to Promote Skeletal Muscle Differentiation and Regeneration,” Genes and Development 28 (2014): 491–501. 24532688 10.1101/gad.234419.113 PMC3950346

[104] Y. Su , Y. Yu , C. Liu , et al., “Fate Decision of Satellite Cell Differentiation and Self‐Renewal by miR‐31‐IL34 Axis,” Cell Death and Differentiation 27 (2020): 949–965. 31332295 10.1038/s41418-019-0390-x PMC7206105

[105] C. G. Crist , D. Montarras , and M. Buckingham , “Muscle Satellite Cells Are Primed for Myogenesis but Maintain Quiescence With Sequestration of Myf5 mRNA Targeted by microRNA‐31 in mRNP Granules,” Cell Stem Cell 11 (2012): 118–126. 22770245 10.1016/j.stem.2012.03.011

[106] P. Dey , M. A. Soyer , and B. K. Dey , “MicroRNA‐24‐3p Promotes Skeletal Muscle Differentiation and Regeneration by Regulating HMGA1,” Cellular and Molecular Life Sciences 79 (2022): 170. 35238991 10.1007/s00018-022-04168-7 PMC11072726

[107] R. Wu , H. Li , L. Zhai , et al., “MicroRNA‐431 Accelerates Muscle Regeneration and Ameliorates Muscular Dystrophy by Targeting Pax7 in Mice,” Nature Communications 6 (2015): 7713. 10.1038/ncomms8713 26151913

[108] K. P. Lee , Y. J. Shin , A. C. Panda , et al., “miR‐431 Promotes Differentiation and Regeneration of Old Skeletal Muscle by Targeting Smad4,” Genes and Development 29 (2015): 1605–1617. 26215566 10.1101/gad.263574.115 PMC4536309

[109] Y. Liu , Y. Yao , Y. Zhang , et al., “MicroRNA‐200c‐5p Regulates Migration and Differentiation of Myoblasts via Targeting Adamts5 in Skeletal Muscle Regeneration and Myogenesis,” International Journal of Molecular Sciences 24 (2023): 4995. 36902425 10.3390/ijms24054995 PMC10003123

[110] L. Zhai , R. Wu , and W. Han , “miR‐127 Enhances Myogenic Cell Differentiation by Targeting S1PR3,” Cell Death and Disease 8 (2017): e2707. 28358363 10.1038/cddis.2017.128 PMC5386531

[111] T. Boettger , S. Wüst , H. Nolte , and T. Braun , “The miR‐206/133b Cluster Is Dispensable for Development, Survival and Regeneration of Skeletal Muscle,” Skeletal Muscle 4 (2014): 23. 25530839 10.1186/s13395-014-0023-5 PMC4272821

[112] M. Nie , J. Liu , Q. Yang , et al., “MicroRNA‐155 Facilitates Skeletal Muscle Regeneration by Balancing Pro‐ and Anti‐Inflammatory Macrophages,” Cell Death and Disease 7 (2016): e2261. 27277683 10.1038/cddis.2016.165 PMC5143393

[113] L. Rodriguez‐Outeiriño , F. Hernandez‐Torres , F. Ramirez de Acuña , et al., “miR‐106b Is a Novel Target to Promote Muscle Regeneration and Restore Satellite Stem Cell Function in Injured Duchenne Dystrophic Muscle,” Molecular Therapy–Nucleic Acids 29 (2022): 769–786. 36159592 10.1016/j.omtn.2022.08.025 PMC9463180

[114] O. Mucha , P. Podkalicka , M. Żukowska , E. Pośpiech , J. Dulak , and A. Łoboda , “miR‐378 Influences Muscle Satellite Cells and Enhances Adipogenic Potential of Fibro‐Adipogenic Progenitors but Does Not Affect Muscle Regeneration in the Glycerol‐Induced Injury Model,” Scientific Reports 13 (2023): 13434. 37596327 10.1038/s41598-023-40729-x PMC10439181

[115] A. Soriano‐Arroquia , R. McCormick , A. P. Molloy , A. McArdle , and K. Goljanek‐Whysall , “Age‐Related Changes in miR‐143‐3p:Igfbp5 Interactions Affect Muscle Regeneration,” Aging Cell 15 (2016): 361–369. 26762731 10.1111/acel.12442 PMC4783349

[116] A. Soriano‐Arroquia , J. Gostage , Q. Xia , et al., “miR‐24 and Its Target Gene Prdx6 Regulate Viability and Senescence of Myogenic Progenitors During Aging,” Aging Cell 20 (2021): e13475. 34560818 10.1111/acel.13475 PMC8520721

[117] Y. Shi , X. Mao , M. Cai , et al., “miR‐194‐5p Negatively Regulates the Proliferation and Differentiation of Rabbit Skeletal Muscle Satellite Cells,” Molecular and Cellular Biochemistry 476 (2021): 425–433. 32997306 10.1007/s11010-020-03918-0 PMC7867548

[118] T. H. Cheung , N. L. Quach , G. W. Charville , et al., “Maintenance of Muscle Stem‐Cell Quiescence by microRNA‐489,” Nature 482 (2012): 524–528. 22358842 10.1038/nature10834 PMC3292200

[119] T. Sato , T. Yamamoto , and A. Sehara‐Fujisawa , “miR‐195/497 Induce Postnatal Quiescence of Skeletal Muscle Stem Cells,” Nature Communications 5 (2014): 4597. 10.1038/ncomms5597 25119651

[120] T. I. Henriksen , P. K. Davidsen , M. Pedersen , et al., “Dysregulation of a Novel miR‐23b/27b‐p53 Axis Impairs Muscle Stem Cell Differentiation of Humans With Type 2 Diabetes,” Molecular Metabolism 6 (2017): 770–779. 28702332 10.1016/j.molmet.2017.04.006 PMC5485225

[121] H. Yin , H. He , X. Cao , et al., “MiR‐148a‐3p Regulates Skeletal Muscle Satellite Cell Differentiation and Apoptosis via the PI3K/AKT Signaling Pathway by Targeting Meox2,” Frontiers in Genetics 11 (2020): 512. 32582277 10.3389/fgene.2020.00512 PMC7287179

[122] A. A. Fiorillo , C. R. Heier , J. S. Novak , et al., “TNF‐α‐Induced microRNAs Control Dystrophin Expression in Becker Muscular Dystrophy,” Cell Reports 12 (2015): 1678–1690. 26321630 10.1016/j.celrep.2015.07.066 PMC4757433

[123] V. Nigro , S. Aurino , and G. Piluso , “Limb Girdle Muscular Dystrophies: Update on Genetic Diagnosis and Therapeutic Approaches,” Current Opinion in Neurology 24 (2011): 429–436. 21825984 10.1097/WCO.0b013e32834aa38d

[124] J. Vissing , “Limb Girdle Muscular Dystrophies: Classification, Clinical Spectrum and Emerging Therapies,” Current Opinion in Neurology 29 (2016): 635–641. 27490667 10.1097/WCO.0000000000000375

[125] C. Bouchard and J. P. Tremblay , “Limb‐Girdle Muscular Dystrophies Classification and Therapies,” Journal of Clinical Medicine 12 (2023): 4769. 37510884 10.3390/jcm12144769 PMC10381329

[126] C. Angelini , R. Marozzo , E. Pinzan , et al., “A New Family With Transportinopathy: Increased Clinical Heterogeneity,” Therapeutic Advances in Neurological Disorders 12 (2019): 1756286419850433. 31217819 10.1177/1756286419850433 PMC6558532

[127] M. Aguennouz , C. Lo Giudice , N. Licata , et al., “MicroRNA Signatures Predict Dysregulated Vitamin D Receptor and Calcium Pathways Status in Limb Girdle Muscle Dystrophies (LGMD) 2A/2B,” Cell Biochemistry and Function 34 (2016): 414–422. 27558075 10.1002/cbf.3202

[128] B. R. R. Nallamilli , S. Chakravorty , A. Kesari , et al., “Genetic Landscape and Novel Disease Mechanisms From a Large LGMD Cohort of 4656 Patients,” Annals of Clinical and Translational Neurology 5 (2018): 1574–1587. 30564623 10.1002/acn3.649 PMC6292381

[129] V. Straub , A. Murphy , and B. Udd , “229th ENMC International Workshop: Limb Girdle Muscular Dystrophies–Nomenclature and Reformed Classification Naarden, the Netherlands, 17‐19 March 2017,” Neuromuscular Disorders 28 (2018): 702–710. 30055862 10.1016/j.nmd.2018.05.007

[130] V. Nigro and M. Savarese , “Genetic Basis of Limb‐Girdle Muscular Dystrophies: The 2014 Update,” Acta Myologica 33 (2014): 1–12. 24843229 PMC4021627

[131] C. Angelini , “LGMD. Identification, Description and Classification,” Acta Myologica 39 (2020): 207–217. 33458576 10.36185/2532-1900-024 PMC7783424

[132] D. G. Georganopoulou , V. G. Moisiadis , F. A. Malik , et al., “A Journey With LGMD: From Protein Abnormalities to Patient Impact,” Protein Journal 40 (2021): 466–488. 34110586 10.1007/s10930-021-10006-9 PMC8190568

[133] C. Angelini , L. Nardetto , C. Borsato , et al., “The Clinical Course of Calpainopathy (LGMD2A) and Dysferlinopathy (LGMD2B),” Neurological Research 32 (2010): 41–46. 20092694 10.1179/174313209X380847

[134] Y. Huang , A. de Morrée , A. van Remoortere , et al., “Calpain 3 Is a Modulator of the Dysferlin Protein Complex in Skeletal Muscle,” Human Molecular Genetics 17 (2008): 1855–1866. 18334579 10.1093/hmg/ddn081 PMC2900895

[135] P. Stuelsatz , F. Pouzoulet , Y. Lamarre , et al., “Down‐Regulation of MyoD by Calpain 3 Promotes Generation of Reserve Cells in C2C12 Myoblasts,” Journal of Biological Chemistry 285 (2010): 12670–12683. 20139084 10.1074/jbc.M109.063966 PMC2857084

[136] S. Hauerslev , M. L. Sveen , M. Duno , C. Angelini , J. Vissing , and T. O. Krag , “Calpain 3 Is Important for Muscle Regeneration: Evidence From Patients With Limb Girdle Muscular Dystrophies,” BMC Musculoskeletal Disorders 13 (2012): 43. 22443334 10.1186/1471-2474-13-43 PMC3338386

[137] J. Lasa‐Elgarresta , L. Mosqueira‐Martín , N. Naldaiz‐Gastesi , et al., “Calcium Mechanisms in Limb‐Girdle Muscular Dystrophy With *CAPN3* Mutations,” International Journal of Molecular Sciences 20 (2019): 4548. 31540302 10.3390/ijms20184548 PMC6770289

[138] F. Magri , L. Napoli , M. Ripolone , et al., “The Profiling of 179 miRNA Expression in Serum From Limb Girdle Muscular Dystrophy Patients and Healthy Controls,” International Journal of Molecular Sciences 24 (2023): 17402. 38139231 10.3390/ijms242417402 PMC10743601

[139] M. Fanin and C. Angelini , “Protein and Genetic Diagnosis of Limb Girdle Muscular Dystrophy Type 2A: The Yield and the Pitfalls,” Muscle and Nerve 52 (2015): 163–173. 25900067 10.1002/mus.24682

[140] C. Paradas , L. González‐Quereda , N. De Luna , et al., “A New Phenotype of Dysferlinopathy With Congenital Onset,” Neuromuscular Disorders 19 (2009): 21–25. 19084402 10.1016/j.nmd.2008.09.015

[141] L. Klinge , J. Harris , C. Sewry , et al., “Dysferlin Associates With the Developing T‐Tubule System in Rodent and Human Skeletal Muscle,” Muscle and Nerve 41 (2010): 166–173. 20082313 10.1002/mus.21166

[142] V. Nigro and G. Piluso , “Spectrum of Muscular Dystrophies Associated With Sarcolemmal‐Protein Genetic Defects,” Biochimica et Biophysica Acta 1852 (2015): 585–593. 25086336 10.1016/j.bbadis.2014.07.023

[143] M. Fanin and C. Angelini , “Defective Assembly of Sarcoglycan Complex in Patients With Beta‐Sarcoglycan Gene Mutations. Study of Aneural and Innervated Cultured Myotubes,” Neuropathology and Applied Neurobiology 28 (2002): 190–199. 12060343 10.1046/j.1365-2990.2002.00389.x

[144] C. Angelini and M. Fanin , “Pathogenesis, Clinical Features and Diagnosis of Sarcoglycanopathies,” Expert Opinion on Orphan Drugs 4 (2016): 1239–1251.

[145] G. N. Maertens , N. J. Cook , W. Wang , et al., “Structural Basis for Nuclear Import of Splicing Factors by Human Transportin 3,” Proceedings of the National Academy of Sciences of the United States of America 111 (2014): 2728–2733. 24449914 10.1073/pnas.1320755111 PMC3932936

[146] M. C. Lai , R. I. Lin , and W. Y. Tarn , “Transportin‐SR2 Mediates Nuclear Import of Phosphorylated SR Proteins,” Proceedings of the National Academy of Sciences of the United States of America 98 (2001): 10154–10159. 11517331 10.1073/pnas.181354098 PMC56931

[147] M. J. Melià , A. Kubota , S. Ortolano , et al., “Limb‐Girdle Muscular Dystrophy 1F Is Caused by a Microdeletion in the Transportin 3 Gene,” Brain 136 (2013): 1508–1517. 23543484 10.1093/brain/awt074 PMC3634201

[148] A. Torella , M. Fanin , M. Mutarelli , et al., “Next‐Generation Sequencing Identifies Transportin 3 as the Causative Gene for LGMD1F,” PLoS One 8 (2013): e63536. 23667635 10.1371/journal.pone.0063536 PMC3646821

[149] A. Vihola , J. Palmio , O. Danielsson , et al., “Novel Mutation in *TNPO3* Causes Congenital Limb‐Girdle Myopathy With Slow Progression,” Neurology: Genetics 5 (2019): e337. 31192305 10.1212/NXG.0000000000000337 PMC6515942

[150] E. Pál , J. Zima , K. Hadzsiev , et al., “A Novel Pathogenic Variant in TNPO3 in a Hungarian Family With Limb‐Girdle Muscular Dystrophy 1F,” European Journal of Medical Genetics 62 (2019): 103662. 31071488 10.1016/j.ejmg.2019.05.001

[151] S. Gibertini , A. Ruggieri , S. Saredi , et al., “Long Term Follow‐Up and Further Molecular and Histopathological Studies in the LGMD1F Sporadic TNPO3‐Mutated Patient,” Acta Neuropathologica Communications 6 (2018): 141. 30567601 10.1186/s40478-018-0648-4 PMC6299540

[152] R. Costa , M. T. Rodia , S. Vianello , et al., “Transportin 3 (TNPO3) and Related Proteins in Limb Girdle Muscular Dystrophy D2 Muscle Biopsies: A Morphological Study and Pathogenetic Hypothesis,” Neuromuscular Disorders 30 (2020): 685–692. 32690349 10.1016/j.nmd.2020.05.006

[153] C. Angelini , V. Pegoraro , and G. Cenacchi , “The Clinical and Molecular Spectrum of Autosomal Dominant Limb‐Girdle Muscular Dystrophies Focusing on Transportinopathy,” Expert Opinion on Orphan Drugs 7 (2019): 223–232.

[154] E. Peterle , M. Fanin , C. Semplicini , et al., “Clinical Phenotype, Muscle MRI and Muscle Pathology of LGMD1F,” Journal of Neurology 260 (2013): 2033–2041. 23632945 10.1007/s00415-013-6931-1

[155] N. O. Mousa , A. Abdellatif , N. Fahmy , H. El‐Fawal , and A. Osman , “MicroRNAs as a Tool for Differential Diagnosis of Neuromuscular Disorders,” Neuromolecular Medicine 25 (2023): 603–615. 37856057 10.1007/s12017-023-08763-0 PMC10721695

[156] W. Wang , T. Li , L. Gao , et al., “Plasma miR‐208b and miR‐499: Potential Biomarkers for Severity of Coronary Artery Disease,” Disease Markers 2019 (2019): 9842427. 31885748 10.1155/2019/9842427 PMC6893238

[157] S. S. Zhou , J. P. Jin , J. Q. Wang , et al., “miRNAS in Cardiovascular Diseases: Potential Biomarkers, Therapeutic Targets and Challenges,” Acta Pharmacologica Sinica 39 (2018): 1073–1084. 29877320 10.1038/aps.2018.30 PMC6289363

[158] M. E. Yalvac , J. Amornvit , C. Braganza , et al., “Impaired Regeneration in Calpain‐3 Null Muscle Is Associated With Perturbations in mTORC1 Signaling and Defective Mitochondrial Biogenesis,” Skeletal Muscle 7 (2017): 27. 29241457 10.1186/s13395-017-0146-6 PMC5731057

[159] I. E. C. Verhaart , K. Putker , D. van de Vijver , et al., “Cross‐Sectional Study Into Age‐Related Pathology of Mouse Models for Limb Girdle Muscular Dystrophy Types 2D and 2F,” PLoS One 14 (2019): e0220665. 31430305 10.1371/journal.pone.0220665 PMC6701749

[160] D. Israeli , J. Cosette , G. Corre , et al., “An AAV‐SGCG Dose‐Response Study in a γ‐Sarcoglycanopathy Mouse Model in the Context of Mechanical Stress,” Molecular Therapy ‐ Methods & Clinical Development 13 (2019): 494–502. 31194043 10.1016/j.omtm.2019.04.007 PMC6545357

[161] A. Cho , “Neuromuscular Diseases: Genomics‐Driven Advances,” Genomics and Informatics 22 (2024): 24. 39593150 10.1186/s44342-024-00027-y PMC11600827

[162] J. A. Lott and P. W. Landesman , “The Enzymology of Skeletal Muscle Disorders,” Critical Reviews in Clinical Laboratory Sciences 20 (1984): 153–190. 6373145 10.3109/10408368409165773

[163] J. P. Mollica , “Skeletal Muscle RNA Extraction in Preparation for RT‐PCR,” Methods in Molecular Biology 630 (2010): 251–260. 20301002 10.1007/978-1-60761-629-0_16

[164] C. C. Pritchard , H. H. Cheng , and M. Tewari , “MicroRNA Profiling: Approaches and Considerations,” Nature Reviews. Genetics 13 (2012): 358–369. 10.1038/nrg3198 PMC4517822 22510765

[165] C. M. Hindson , J. R. Chevillet , H. A. Briggs , et al., “Absolute Quantification by Droplet Digital PCR Versus Analog Real‐Time PCR,” Nature Methods 10 (2013): 1003–1005. 23995387 10.1038/nmeth.2633 PMC4118677

